# Synthesis, molecular docking and molecular dynamics simulations, drug-likeness studies, ADMET prediction and biological evaluation of novel pyrazole-carboxamides bearing sulfonamide moiety as potent carbonic anhydrase inhibitors

**DOI:** 10.1007/s11030-024-10901-0

**Published:** 2024-06-13

**Authors:** İrfan Yetek, Samet Mert, Ekrem Tunca, Alpaslan Bayrakdar, Rahmi Kasımoğulları

**Affiliations:** 1 Department of Chemistry, Faculty of Arts and Sciences, Dumlupınar University, Kütahya, 43100 Türkiye; 2 Department of Biochemistry, Faculty of Arts and Sciences, Dumlupınar University, Kütahya, 43100 Türkiye; 3 Vocational School of Higher Education for Healthcare Services, Iğdır University, Iğdır, 76000 Türkiye

**Keywords:** Pyrazole-carboxamide, Sulfonamide, Inhibition, Carbonic anhydrase, Molecular docking

## Abstract

Pyrazoles are unique bioactive molecules with a versatile biological profile and they have gained an important place on pharmaceutical chemistry. Pyrazole compounds containing sulfonamide nuclei also attract attention as carbonic anhydrase (CA) inhibitors. In this study, a library of pyrazole-carboxamides were synthesized and the structures of the synthesized molecules were characterized using FT-IR, ^1^H-NMR, ^13^C-NMR and HRMS. Then the inhibition effects of newly synthesized molecules on human erythrocyte hCA I and hCA II isoenzymes were investigated. *K*_i_ values of the compounds were in the range of 0.063–3.368 µM for hCA I and 0.007–4.235 µM for hCA II. Molecular docking studies were performed between the most active compounds ***6a***, ***6b*** and the reference inhibitor, acetazolamide (***AAZ***) and the hCA I and hCA II receptors to investigate the binding mechanisms between the compounds and the receptors. These compounds showed better interactions than the ***AAZ***. ADMET analyzes were performed for the compounds and it was seen that the compounds did not show AMES toxicity. The stability of the molecular docking results over time was analysed by 50 ns molecular dynamics simulations. Molecular dynamics simulations revealed that ***6a*** and ***6b*** exhibited good stability after docking to the binding sites of hCA I and hCA II receptors, with minor conformational changes and fluctuations.

**Graphical abstract:**

Synthesis, molecular docking, molecular dynamics simulations, drug-likeness, ADMET prediction and biological evaluation of pyrazole-carboxamides bearing sulfonamide moiety as potent carbonic anhydrase inhibitors

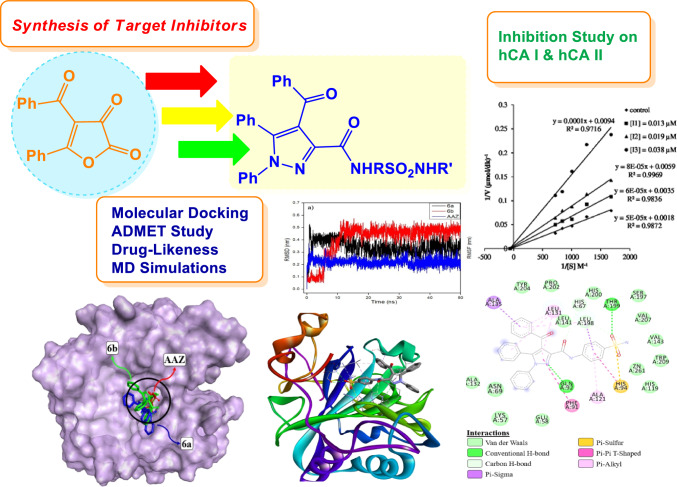

**Supplementary Information:**

The online version contains supplementary material available at 10.1007/s11030-024-10901-0.

## Introduction

**Fig. 1:**
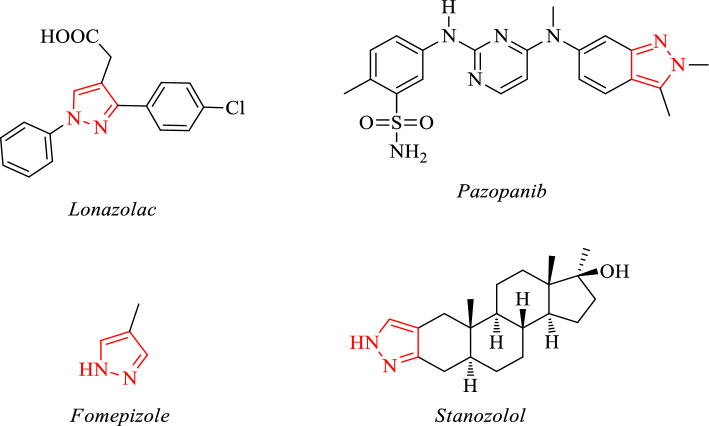
Some examples of pyrazole based drugs, antidotes and steroids

Pyrazoles, a five membered heterocycle containing two adjacent nitrogen atoms, are the core structures widely found in a number of molecules that exhibit a diverse array of chemical and biological properties [1–5]. Besides, pyrazole compounds attract attention especially due to their agrochemical and some pharmaceutical activities [6]. For instance, pyrazoles have pharmaceutical activities in many specific areas, such as antifungal, antitumor, antiinflammatory, anticonvulsant, antiobesity and DNA gyrase inhibitors, among others and they have found application in agrochemistry especially in crop protection [7–14]. Up to now, several pyrazole derivatives have been developed and commercialized as medicines. Among them, Lonazolac is a nonsteroidal anti-inflammatory drug (NSAID), Pazopanib is a cancer medicine using in the treatment of kidney cancer (advanced renal cell carcinoma), Fomepizole an inhibitor of alcohol dehydrogenase used as antidote in methanol or ethylene glycol poisoning and Stanozolol used in the treatment of aplastic anemia and hereditary angioedema [15–19] (**See **Fig. 1).

Carbonic anhydrase (CA) enzymes play a role in many physiological/metabolic events in mammalians such as pH and CO_2_ homeostasis, respiration, CO_2_/bicarbonate transport, electrolyte release in tissues, gluconeogenesis, lipogenesis, ureagenesis, bone resorption, calcification and tumorigenicity [20]. Since human CA isoforms (hCAs) play important roles in the physiological/metabolic events mentioned above, abnormal levels or activities of these isoforms are associated with a number of disorders such as retinal/cerebral edema [21], glaucoma [22–24], epilepsy [25–27], altitude sickness [28], stroke [29], obesity [30], cancer [31, 32], carcinogenesis [33], retinitis pigmentosa [34], sterility [35], retinopathy [36]. For all these reasons, hCAs have been the target of inhibitors/activators used in therapeutic or diagnostic applications. Although a number of molecules have been designed, synthesized and tested on hCAs, there is still a major barrier to their becoming drugs due to their limited selectivity for a specific CA isoform. Until isoform-selective inhibitors/activators are synthesized, the need for new CA agents will continue. Aromatic and heterocyclic scaffolds containing sulfonamide group have been extensively studied to obtain potent and highly selective agents [37–39]. However, studies examining the CA inhibition potentials of pyrazole-sulfonamide derivatives together with complementary molecular dynamics simulation studies are limited in the literature. In a previous study performed by our group, nitrophenyl and aminophenyl substituted pyrazole-dicarboxamide derivatives were synthesized and their CA inhibitory properties were examined in vitro [22]. So the present study novel phenyl substituted pyrazole-carboxamide derivatives carrying sulfonamide moiety (***6a*–*i***) were synthesized and the inhibition effects of these molecules on human erythrocyte hCA I and hCA II isoenzymes were investigated. In addition, the inhibition effects of the compounds with the best activity on hCA I and hCA II isoenzymes were examined by molecular docking, molecular dynamics simulation studies and the results were compared with the reference inhibitor, acetazolamide (***AAZ***). In addition, drug similarity properties were examined according to Lipinski’s five criteria.

## Results and discussion

### Chemistry

In general, furandiones are considered suitable and versatile synthons in heterocyclic chemistry and have proven to be useful agents for the synthesis of pyrazole-3-carboxylic acids [40, 41]. Herein this one-step method were used and 4-benzoyl-1,5-diphenyl-1*H*-pyrazole-3-carboxylic acid (***3***), our starting compound, were obtained via the reaction of 4-benzoyl-5-phenylfuran-2,3-dione (***1***) with hydrazone (***2***) under heating in a solventless media. Pyrazole carboxylic acid ***3***, was converted to acid chloride (***4***) reacting with excess SOCl_2_ in a solventless media again. Starting compounds are not novel and, their characterization was previously made in the literature [40–42].

**Scheme 1:**
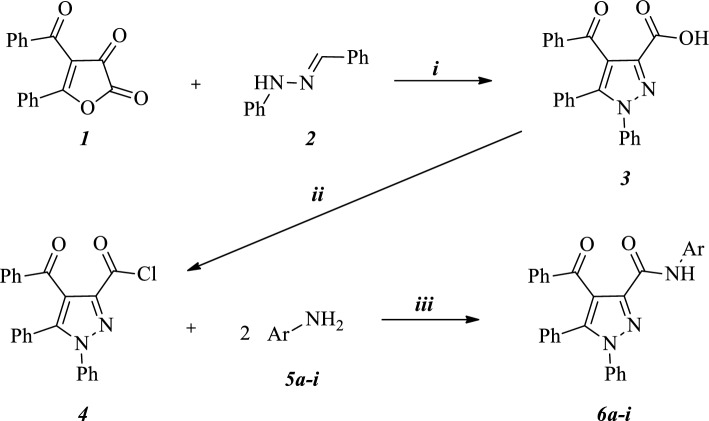
Sythesis of the target compounds (***i***: heat, 80 °C, 2 h, solvent-free; ***ii***: SOCl_2_, reflux, 80 °C, 5 h; ***iii***: 2 eq Sulfonamide derivative, THF, reflux, 5 h)

In last step, novel pyrazole-carboxamides (***6a*–*i***) were synthesized from the reaction of ***4*** with various sulfonamide derivatives (***5a*–*i***). Acid chloride (***4***) was reacted with sulfonamide derivatives (***5a*–*i***) in the molar range of 1:2 in THF under reflux for 5 h to give the target products (***6a*–*i***) in high yields (See Scheme 1). The structures of all newly synthesized compounds were confirmed by spectroscopic data such as FT-IR, ^1^H-NMR, ^13^C-NMR and HRMS analysis.

The infrared spectra (FT-IR) of all synthesized pyrazole-carboxamide derivatives (***6a*–*i***) showed NH stretching vibrations at 3427‒3224 cm^−1^. SO_2_ asymmetric and symmetric stretching vibrations were observed in the range of 1397‒1320 and 1167‒1144 cm^−1^, respectively. Therefore, the IR spectrum results support the presence of sulfonamide groups in the synthesized compounds. Primary/secondary sulfonamides were preferred for amidation reactions. In the ^1^H-NMR spectra of pyrazole-carboxamides (***6a*–*i***) CONH protons observed between *δ* = 10.94‒10.76 ppm. In the ^13^C-NMR spectra of ***6a*–*i***, ketone carbonyl groups were observed in the range of *δ* = 191.23‒191.07 ppm, while amide carbonyl groups were appeared in the range of *δ* = 162.78‒159.00 ppm. In addition, -SO_2_NH_2_ and -SO_2_NH- protons gave signals in different places in the ^1^H-NMR spectra. For example, in amides containing primary sulfonamide (***6a*–*c***), the -SO_2_NH_2_ protons were observed in the range of *δ* = 7.79‒7.37 ppm, while in amides containing secondary sulfonamide -SO_2_NH protons adjacent to a heterocycle (***6d*–*i***) were observed in the range of *δ* = 12.71‒10.96 ppm. Signals for the aromatic protons were observed in the range of *δ* = 7.96‒7.16 ppm. As a result, we can say that the characteristic signals were consistent within themselves in molecules containing the same sulfonamide derivative.

In the ^1^H-NMR spectrum of ***6d*** containing sulfapyridine ring, the doublet signal observed at *δ* = 8.01 ppm indicates the proton attached to the C-6 carbon (C6-H) of the pyridine ring, while a triplet signal at *δ* = 7.55 ppm belongs to the C4-H proton in the same ring. The C3-H proton of the pyridine ring seen as a doublet at *δ* = 7.13 ppm, while the C5-H proton in the same ring observed as a triplet at *δ* = 6.86 ppm. Considering the ^13^C-NMR spectrum values of ***6d***, C-2 carbon of the pyridine ring observed at *δ* = 159.95 ppm, while C-6 and C-3 carbons of the pyridine ring appeared at *δ* = 153.45 and *δ* = 114.03 ppm, respectively. In the ^1^H-NMR spectrum of ***6e*** containing sulfadiazine ring, the C4-H and C6-H protons in the pyrimidine ring observed as 2H doublets at *δ* = 8.50 ppm, while the C5-H proton appeared as triplet at *δ* = 7.04 ppm. According to the ^13^C-NMR spectrum values of the same molecule, it is seen that the quaternary carbon (C-2) in the pyrimidine ring between the two nitrogen atoms observed at *δ* = 158.82 ppm. C-4 and C-6 carbons in the same ring neighboring to nitrogen atoms observed at *δ* = 157.38 ppm, while C-5 carbon not adjacent to nitrogen atom appeared at *δ* = 116.28 ppm. In the ^1^H-NMR spectrum of ***6f*** containing sulfamerazine, the C6-H proton of the pyrimidine ring observed as a doublet at *δ* = 8.31 ppm, while the C5-H proton of the same ring seen as a doublet at *δ* = 6.90 ppm. When the ^13^C-NMR spectrum values of the same molecule were examined, the pyrimidine C-2 and C-4 carbons observed at *δ* = 169.38 and *δ* = 162.79 ppm, respectively. Also the C-6 and C-5 carbons in the same ring observed at *δ* = 157.01 and *δ* = 100.01 ppm, respectively. Again, the methyl group attached to the 4 position of the same ring observed at *δ* = 23.75 ppm. When the ^1^H-NMR spectrum of ***6g*** were analyzed which containing sulfisoxazole, the methyl protons bonded to the third and fourth positions of the isoxazole ring appeared as single peaks at *δ* = 2.08 ppm and *δ* = 1.63 ppm, respectively. In addition, in the ^13^C-NMR spectrum of ***6g***, C-3 and C-5 carbons of the isoxazole ring were observed at *δ* = 161.89 and *δ* = 155.99 ppm, respectively, while the C-4 carbon not adjacent to the heteroatom was observed at *δ* = 105.58 ppm. Signals of the methyl groups attached to the third and fourth positions of the isoxazole ring observed at *δ* = 10.81 and 6.35 ppm, respectively. When the ^1^H-NMR spectrum of ***6h*** were examined which containing sulfathiazole, the thiazole ring C4-H and C5-H protons appeared as doublets at *δ* = 7.18 and *δ* = 6.81 ppm, respectively. When the ^13^C-NMR spectrum values of the same molecule were examined, the C-2 and C-4 carbons of the thiazole ring gave signals at *δ* = 169.23 and *δ* = 137.44 ppm, respectively. Also the C-5 carbon in the same ring were observed at *δ* = 108.59 ppm. When the ^1^H-NMR spectrum of ***6i*** were examined which containing sulfaguanidine, the NH and NH_2_ protons in the compound were observed as broad singlet at *δ* = 6.67 ppm. Looking at the ^13^C-NMR spectrum values, the guanidine group carbon atom (C = NH) was observed at *δ* = 158.52 ppm. As a result, the spectral values of all pyrazole-carboxamides (***6a*–*i***) synthesized from amidation reactions were in full harmony with the targeted molecules, indicating that the reactions have taken place successfully.

### In vitro carbonic anhydrase inhibition of the compounds

**Fig. 2:**
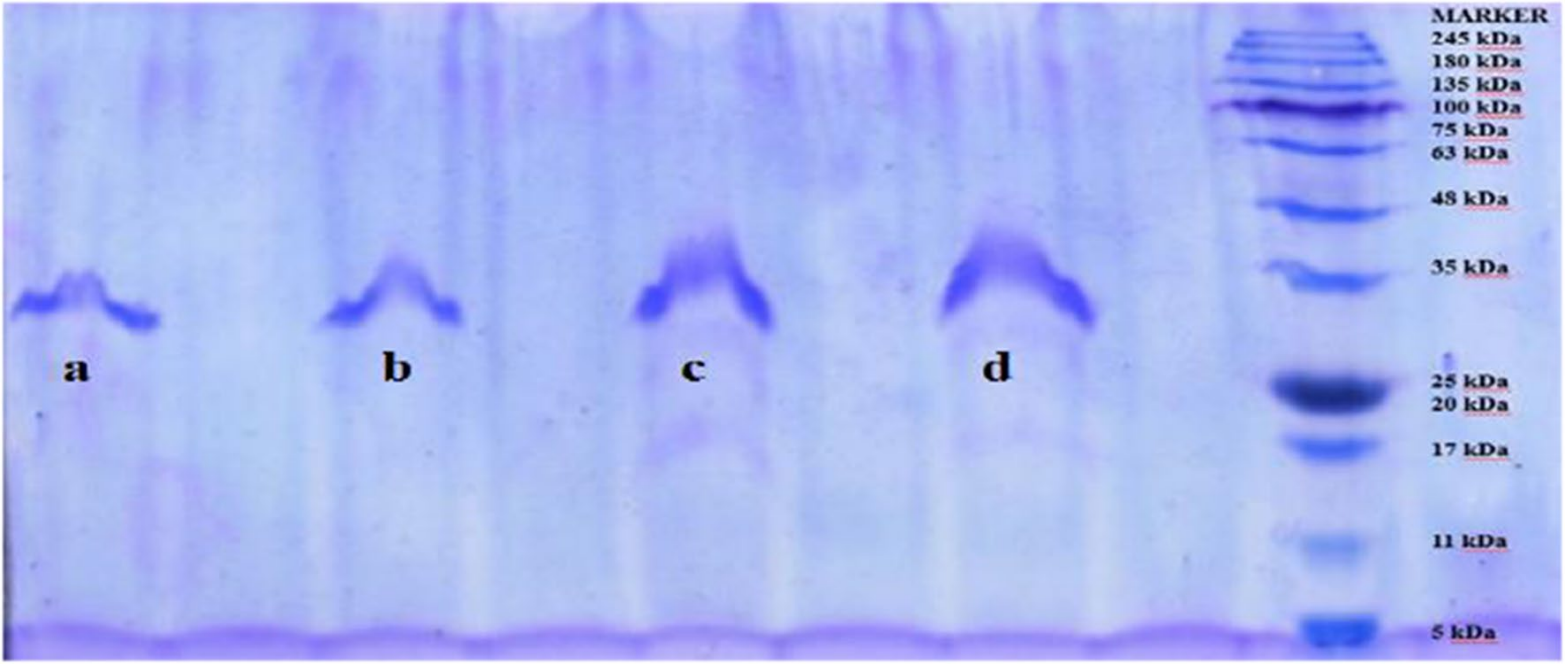
SDS-PAGE analysis of purified hCA I (**a**, **b**) and hCA II (**c**, **d**)

To examine the inhibitory effects of the synthesized compounds on hCA I and hCA II, these isoenzymes have purified by affinity chromatography (See Table 1). SDS-PAGE analysis have confirmed that hCA I and hCA II have obtained in high purity (See Fig. 2).

**Table 1:** Purification data of hCA I and hCA II isozymes

Purification Steps		Activity (EU/ml)	Total Volume (ml)	Protein (mg/ml)	Total Protein (mg)	Total Activity (EU)	Specific Activity (EU/mg protein)	Yield (%)	Purification Factor
Hemolysate		148.67	100	19.94	1994	14867	7.46	100	1
Affinity column	hCA I	462.85	5	0.45	2.25	2314.25	1028.56	15.57	137.88
	hCA II	603.74	5	0.38	1.90	3018.7	1588.79	20.31	212.97

When the inhibition profile of esterase activities of hCA I and hCA II is examined in terms of esterase IC_50_ values, it is striking that compounds with free sulfonamide group show higher inhibition than those without. Moreover, the inhibition potential of the compounds appears to vary significantly with the position of the free sulfonamide group.

**Fig. 3:**
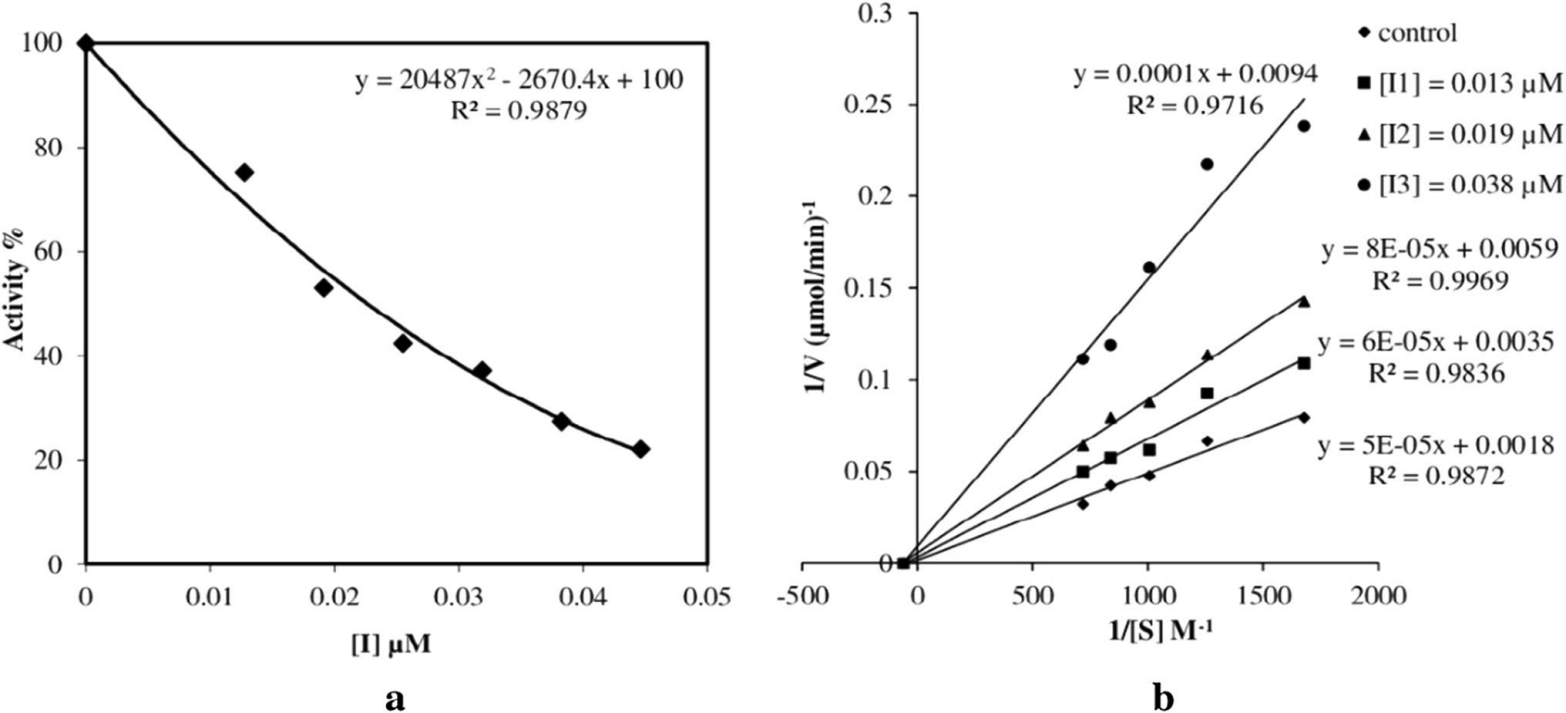
Esterase IC_50_ (**a**) and *K*_i_ (**b**) graphs of ***6a*** for hCA II

To examine in more detail, ***6a***, which carries the primary -SO_2_NH_2_ in the *para*- position, is the strongest esterase inhibitor for both hCA I and hCA II. In terms of inhibition potential, compound ***6a*** has followed by ***6b*** containing the primary -SO_2_NH_2_ group in the *meta*- position. The third compound in the ranking is not ***6c*** as expected. There is also a free -SO_2_NH_2_ group in compound ***6c***, but since this group is in the *ortho*—position, it could not interact with Zn^2+^ in active site strongly enough due to the steric hindrance. When IC_50_ values are examined, it is seen that compound ***6g*** ranks third in terms of esterase inhibition potential. The lone-pair electrons on the oxygen atom in the 3,4-dimethylisoxazole group in this compound may have interacted more efficiently with the active site. Compound ***6h*** with thiazole in its structure exhibited a very weak inhibition effect for hCA I. The same compound did not inhibit the hCA II isoenzyme. This shows that the thiazole group does not interact with Zn^2+^, but interacts with other amino acids in the active site. Among the compounds containing secondary sulfonamide, compound ***6d*** with pyridine, ***6e*** with pyrimidine, ***6f*** with 4-methylpyrimidine and ***6i*** with guanidine did not show any inhibition effect on both hCA I and hCA II. It is estimated that the electron density on the nitrogen atoms in the above-mentioned groups in these compounds (***6d***, ***6e***, ***6f*** and ***6i***) is not sufficient to interact with the active site (**See **Table 2 and Fig. 3).

**Table 2:** Inhibition data of hCA I and hCA II

Compound		Esterase IC_50_ (μM)		K_i_ (μM)			
		hCA I	hCA II	hCA I	Inhibition Type	hCA II	Inhibition Type
	Ar						
AAZ		0.420	0.310	0.260	Non competitive	0.140	Non competitive
6a		0.182	0.023	0.063	Non competitive	0.007	Non competitive
6b		0.463	0.139	0.237	Non competitive	0.030	Non competitive
6c		6.204	6.410	3.368	Non competitive	4.235	Non competitive
6d		NI	NI	NI	–	NI	–
6e		NI	NI	NI	–	NI	–
6f		NI	NI	NI	–	NI	–
6g		1.660	0.705	NI	–	0.365	Non competitive
6h		57.988	NI	NI	–	NI	–
6i		NI	NI	NI	–	NI	–

Mean from three different assays, by a spectrophotometric technique (errors were in the range of ± 5‒10% of reported values)

*p* < 0.0001 for all analysis

*AAZ* was used as reference compound

*NI* No Inhibition

When the *K*_i_ values of the compounds are examined, although there is a similar inhibition order, there are some important differences. The *K*_i_ value for hCA I of compound ***6g*** could not calculated. Because this compound was able to decrease the activity of the hCA I isoenzyme by 50% at most. However, the same compound has a good *K*_i_ value for hCA II. The fact that the hCA I *K*_i_ value of compound ***6h*** could not be calculated is based on the same reason.

When the isoform selectivities of the compounds are evaluated, it is clear that compounds ***6a***, ***6b*** and ***6g*** are more selective for hCA II. In particular, ***6g*** could not exhibit over 50% inhibition potential for hCA I. However, it has a *K*_i_ value that can considered effective for hCA II. Comparing with the reference compound for hCA II, ***6a*** has a 20-fold stronger inhibitory effect, and ***6b*** has approximately fivefold stronger inhibitory effect than ***AAZ***. In addition, our compounds are compatible with the “tail approach” strategy, which allows selective interaction with the active site of CA isoenzymes. In particular, the phenyl groups attached to the pyrazole ring have the potential to interact with the Phe131 residue in hCA II. The fact that most of the compounds showing inhibitory effect are more selective for hCA II confirms this situation.

### Docking studies

The most active compounds interacting with the receptors in in vitro studies were determined as ***6a*** and ***6b***. In the in silico part of this study, docking studies were performed to determine the best binding pose and possible mechanism of inhibition of the most active compounds to hCA I (2CAB) and hCA II (1CA2) receptors. ***AAZ*** was used as the reference inhibitor for hCAI and hCAII receptors in the molecular docking study. In the docking study, the most active compounds and the ***AAZ*** reference compound were docked to the active site of the receptors.

**Fig. 4:**
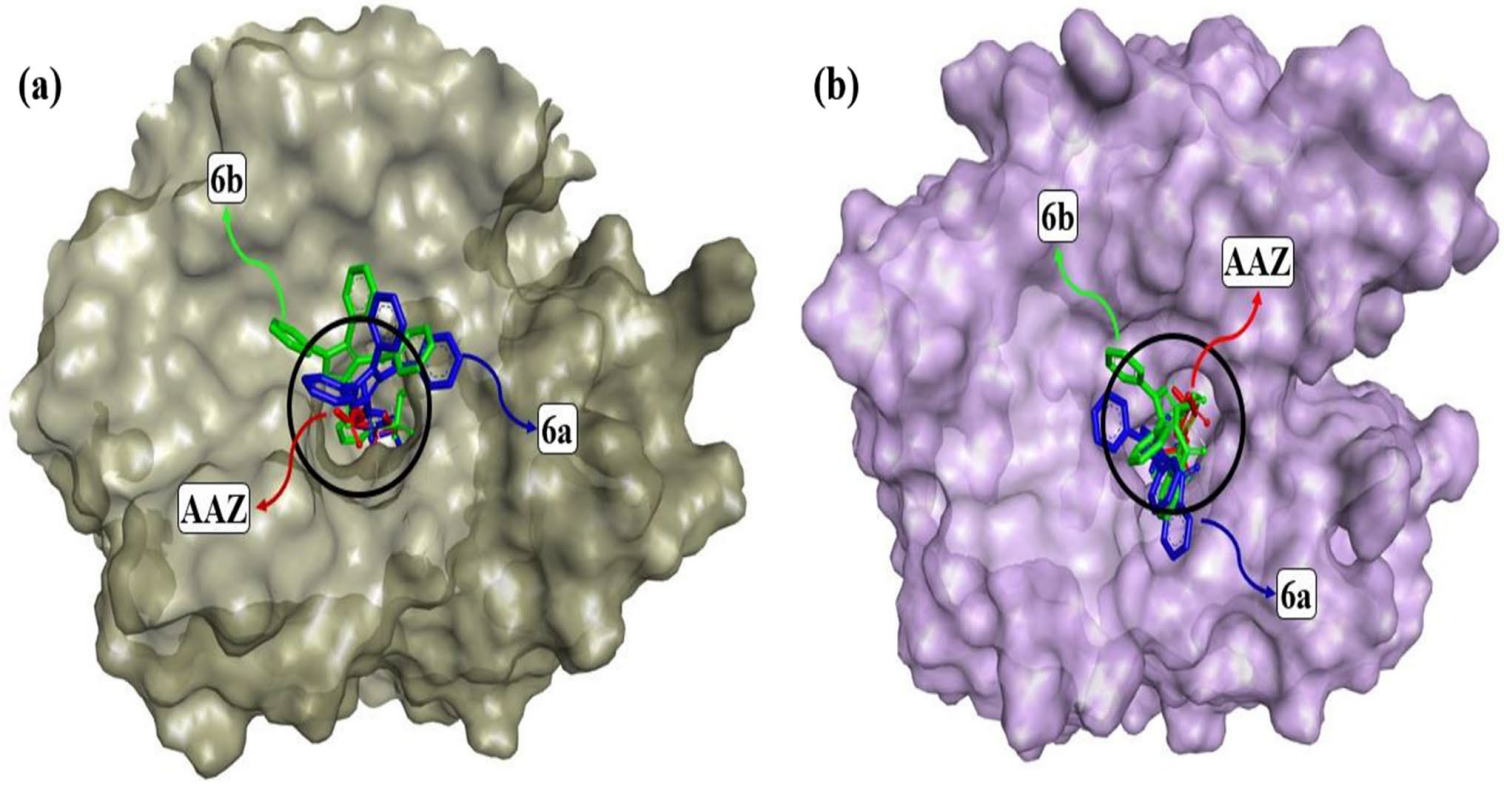
Protein–ligand docking conformations. Receptors hCA I (grey color) and hCA II (light purple color) are shown in solid surface model. The best exposures of ligands ***6a***, ***6b*** and ***AAZ*** docked on the receptors are represented by the colors blue, green and red, respectively, in stick modeling

The docking study resulted in multiple binding pose for the newly synthesized compounds that were inhibitor candidates. Analysis of interactions between the most active compounds and receptors was performed using the BIOVIA Discovery Studio Visualizer software. As a result of the molecular docking study, the best calculated poses of the inhibitor candidate compounds that docking with hCA I and hCA II receptors, respectively, are shown in Fig. 4a (grey) and Fig. 4b (light purple). Figure 4 clearly demonstrated that compounds ***6a***, ***6b*** and ***AAZ*** gradually docked to the active site of both hCA I and hCA II receptors. In the molecular docking study, the docking mechanisms of the hCA I@***6a***, hCA I@***6b***, hCA I@***AAZ***, hCA II@***6a***, hCA II@***6b*** and hCA II@***AAZ*** complexes formed between amino acid residues in the active site of the hCA I and hCA II receptors and compound ***6a***, ***6b*** and the ***AAZ*** are summarized in Tables 3 and 4.

**Table 3:** Summative results of the molecular docking of the most active compunds and reference inhibitor into hCA I

Ligands	Binding score (kcal/mol)	Hydrogen Bond interactions (Å)	Hydrophobic interaction (Å)	Electrostatic and other interaction (Å)	Van der Waals
*AAZ*	− 6.0	Conventional H-Bond Ser193(2.70), Thr195(2.27) Carbon H-Bond His90(2.76)	Pi-Pi T-shaped His90(5.16) Pi-Alkyl Leu194(4.10)	Metal Acceptor Zn(3.02) Pi-sulfur His90(5.06–5.14)	Phe87, Gln88, His115, Ala117, Leu127, Leu137, Val139, His196, Val203
*6a*	− 9.3	Conventional H-Bond Thr199(2.87–2.88), Gln92(2.95) Carbon H-Bond His67(3.23), Leu198(3.28)	Pi-Sigma Ala135(3.78), Leu198(3.28) Pi-Pi T-shaped His94(4.54), Phe91(5.69) Pi-Alkyl Leu131(5.26–5.42–5.50), Ala121(4.97), Leu198(5.01)	Pi-Sulfur His94(4.97) His94-Zn(1.93)	Lys57, Glu58, His67, Asn69, His119, Ala121, Ala132, Leu141, Val143, Ser197, His200, Pro202, Tyr204, Val207, Trp209
*6b*	− 7.6	Conventional H-Bond Thr199(2.77–2.90–3.04), Gln92(2.48), His200(3.26) Carbon H-Bond His67(2.82) Pi-Donor H-Bond Gln92(3.42)	Pi-Sigma Leu198(5.49), Pi-Pi T-shaped His94(5.24), His200(5.92) Pi-Alkyl Val62(5.00), Val143(5.33), Ala121(4.32)	Metal Acceptor Zn(2.46) Pi-Sulfur His96(5.79)	AlaTrp5, His64, Ile60, Asn69, Phe91, Leu131, Leu141, Val207, Trp209

**Table 4:** Summative results of the molecular docking of the most active compunds and reference inhibitor into hCA II

Ligands	Binding score (kcal/mol)	Hydrogen Bond interactions (Å)	Hydrophobic interaction (Å)	Electrostatic and other interaction (Å)	Van der Waals
*AAZ*	− 6.1	Conventional H-Bond Asn67(2.52), Thr199(3.11–2.73), Thr200(2.70–3.06) Pi-Donor H-Bond Thr200(3.76)	Pi-Pi Stacked His94(4.02)	Metal Acceptor Zn(2.39) Pi-sulfur His96(5.72)	Asn62, His64, Gln92, His119, Val143, Trp209, Leu198
*6a*	− 8.5	Conventional H-Bond Thr199(2.82–3.05), Gln92(3.05) Carbon H-Bond Leu198(2.88)	Pi-Sigma Ile91(3.89), Leu198(3.51) Pi-Pi Stacked Phe131(4.54) Pi-Pi T-shaped His94(4.54) Pi-Alkyl Val121(4.98), Val135(4.7), Pro202(4.13)	Metal Acceptor Zn(2.34) Pi-Sulfur His94(5.04)	Asn67, His96, His119, Ser197, Thr200, Val207, Trp209
*6b*	− 7.9	Conventional H-Bond Thr199(2.77–3.05), Thr200(2.70–3.02), Asn62(2.93)	Pi-Alkyl Ile91(5.28), Val135(5.33), Leu198(5.45), Pro202(5.14) Pi-Sigma Ile91(3.93) Pi-Pi stacked His94(3.82) Pi-Pi T-shaped Phe131(5.03)	Metal Acceptor Zn(2.46) Pi-Anion Glu69(3.99)	Asn62, His64, Ala65, Asn67, His96, His119, Val121, Val143

For the analysis of the docking results, we took into account Binding score change, hydrogen bonds and non-bonded interactions. Binding score of the complexes were calculated considering the interaction types of ***6a***, ***6b*** and ***AAZ*** with amino acid residues of hCA I and hCA II receptors, as shown in Tables 3 and 4, respectively. The binding score values for the complexes were calculated as -9.3 for hCA I@***6a***, -8.5 for hCA II@***6a***, -7.6 for hCA I@***6b***, -7.9 for hCA II@***6b***, -6.0 for hCA I@***AAZ*** and -6.1 for hCA II@***AAZ***. The results clearly showed that the most active compounds had higher binding affinities for hCA I and hCA II receptors compared to the ***AAZ***.

**Fig. 5:**
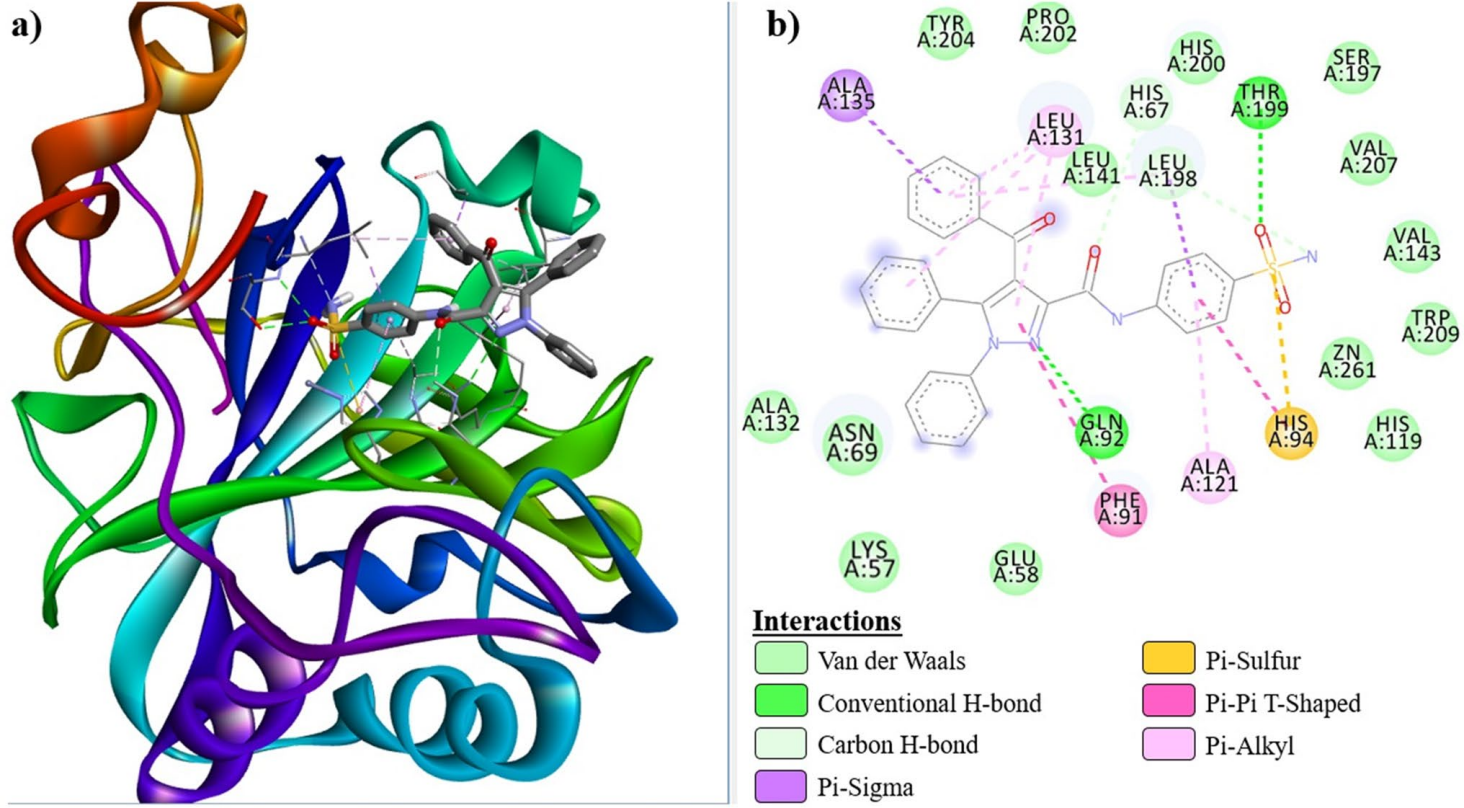
hCA I@***6a*** receptor-ligand interaction diagrams; **a** 3D, **b** 2D

**Fig. 6:**
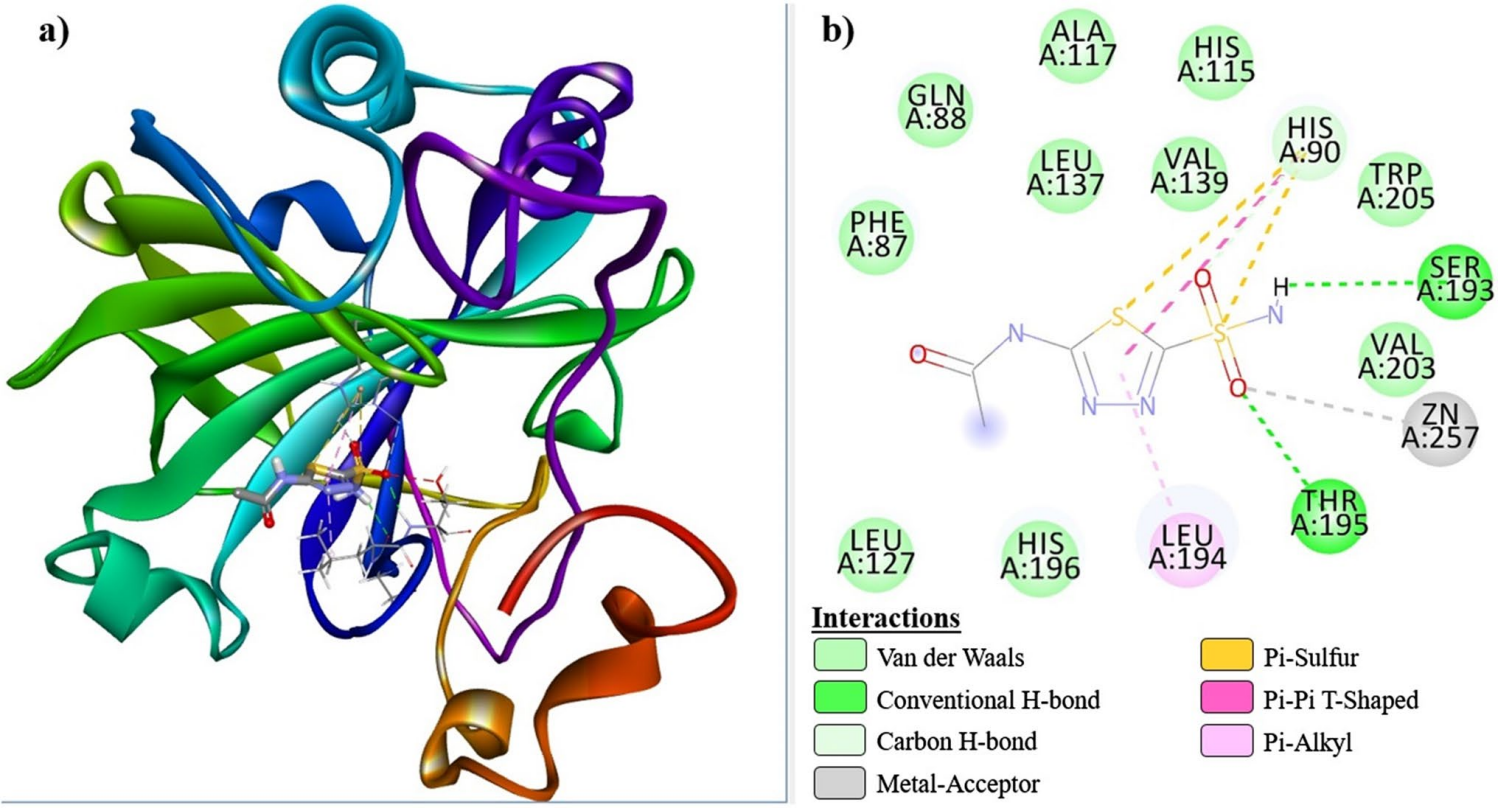
hCA I@***AAZ*** receptor-ligand interaction diagrams; **a** 3D, **b** 2D

**Fig. 7:**
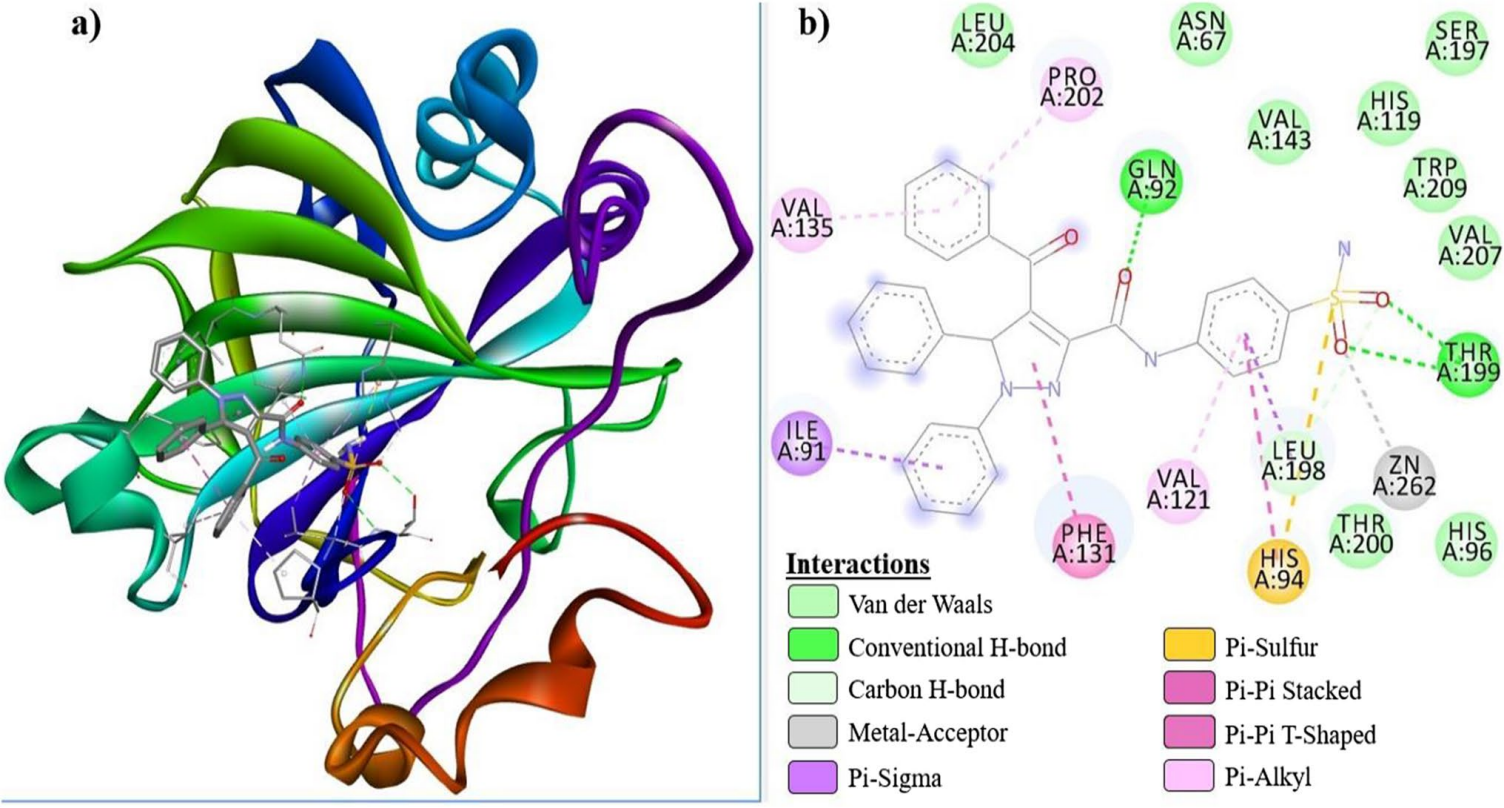
hCA II@***6a*** receptor-ligand interaction diagrams; **a** 3D, **b** 2D

**Fig. 8:**
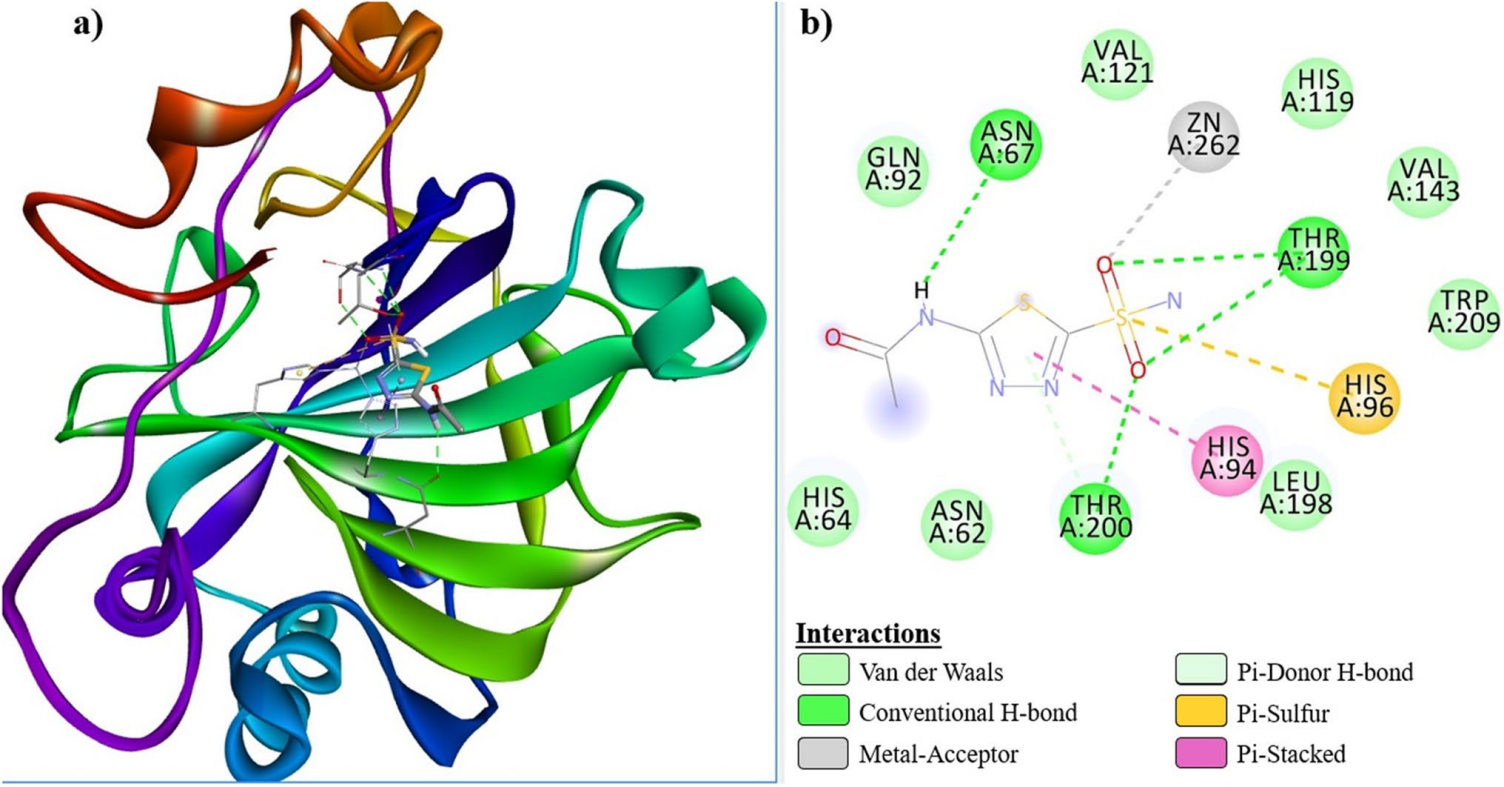
hCA II@***AAZ*** receptor-ligand interaction diagrams; **a** 3D, **b** 2D

The binding mechanisms of 2D and 3D receptor-ligand interactions of compound ***6a***, which is the best candidate among the compounds in terms of binding Δ, and the control compound ***AAZ*** were investigated in detail. In this context, 3D and 2D receptor-ligand interaction diagrams of hCA I@***6a***, hCA I@***AAZ***, hCA II@***6a*** and hCA II@***AAZ*** complexes are shown in Figs. 5, 6, 7, 8, respectively. The inhibition of hCA receptors relies on various interactions between ligands and active residues, including H-Bond, Pi-Cation, Pi-Sigma, Pi-Alkyl, Pi-Pi T-Shaped, Van der Waals, Pi-sulfur, and Metal-acceptor bond interactions with Thr199, His94, His96, His119, and His200. The docking results presented in Tables 3 and 4 indicate that all necessary interactions for inhibition were present in the formed complexes. Tables 3 and 4 present the docking results summary, which indicates that all the interactions necessary for inhibition were present in the formed complexes.

Compounds ***6a***, ***6b***, and ***AAZ*** were strongly docked to the active site of hCA I via H-bond and hydrophobic interactions. Furthermore, it formed a Pi-sulfur bond with His94. Compound ***6a*** interacted with Zn^+2^ via His94, to which it was connected by a Pi-sulfur bond, while ***AAZ*** and ***6b*** performed metal-acceptor bond interactions. Compounds ***6a*** and ***6b*** formed Pi-sulfur bonds with His94 and His96, respectively, while ***AAZ*** interacted with His90 in the active site.

Similarly, compounds ***6a***, ***6b*** and ***AAZ*** were strongly localized to the active site of hCA II through H-bond and hydrophobic interactions. All three compounds formed metal-acceptor bond interactions with the Zn^+2^ ion in the active site. ***AAZ*** and ***6a*** interacted with His96 and His94 via a Pi-sulfur bond, while ***6b*** formed a Pi-anion bond with Glu69. Compound ***6a***, which has the ‒SO_2_NH_2_ group in the para position, oriented towards the Zn^+2^ ion located at the bottom of the active site, as expected, during its interaction with hCA I and hCA II receptors. It is predictable that the angularly suitable phenyl substituents in the structure of this compound will interact with Phe91, Leu131, Ala135, and Leu141, which are apolar amino acid residues at the entrance of the active site of the hCA I receptor. The same compound interacted with Ile91, Phe131, Val135, and Pro202, which are apolar residues at the active site entrance of the hCA II receptor. Especially the fact that residues Phe131 and Val135 are more bulky in the hCA II receptor increased the receptor-ligand interaction and enabled a stronger inhibition of this enzyme. These interactions are clearly seen in Figs. 5b and 7b.

Figure 4a, b show the conformations of ***6a***, ***6b*** and ***AAZ*** in the complexes they form with hCA I and hCA II receptors, respectively. It has been reported that ligand interactions with the Zn^+2^ ion in the active site of hCA enzymes enhance enzyme inhibition [43]. Tables 3 and 4, which summarize the receptor-ligand interactions, clearly show that the sulfonamide group without steric hindrance in the compounds ***6a*** and ***6b*** contributes to the inhibition by interacting with the Zn^+2^ ion, which, similar to ***AAZ***, is located in the active site of the receptors and is associated with enzyme activity. This situation is also clearly seen in the 2D interaction diagrams of compounds ***6a*** and ***AAZ*** given in Figs. 5, 6, 7, 8.

### ADMET study

The ADMET and drug-likeness estimates made through the web-based online pkCSM tool for ***6a***, ***6b*** and ***AAZ*** are summarized in Tables 5 and 6. Toxicity analysis is one of the important and guiding methods used in faster and cheaper new drug designs. The log *K*p value in Table 5 showed that the compounds exhibited very good gastrointestinal drug absorption and a low skin permeability (log *K*p > ‒ 2.5). VDss, a pharmacokinetic parameter, is a measure of the distribution of the drug in the body [44]. When Table 5 is examined, the VDss values of the compounds are low. This means low concentration in tissues for all compounds. When the AMES toxicity values of the compounds were examined in the table, it was understood that they did not show any toxicity or mutagenic effect. Hepatotoxicity refers to the damage caused to the liver by substances such as food or drugs that enter the body. Predictions about the hepatotoxicity of the compounds were made in the ADMET study and provided in the Table 5. The skin sensitization predictions of the compounds indicated that there is no allergic condition associated with the compounds. The HIA values were calculated as 85.328, 83.593, and 54.854% for compounds ***6a***, ***6b***, and ***AAZ***, respectively. These values showed that the most active compounds were less absorbed in the human body compared to ***AAZ***.

**Table 5:** ADMET analysis results obtained by using pkCSM tools

	Compounds		
	*6a*	*6b*	*AAZ*
*Absorption*			
Water solubility (log mol/L)	− 4.782	− 4.821	− 1.925
Caco2 permeability (log Papp in 10^−6^ cm/s)	0.717	0.718	− 0.305
Human intestinal absorption (HIA + , %)	85.328	88.299	54.854
Skin permeability (log *K*p)	− 2.827	− 2.812	− 2.736
P-glycoprotein substrate	Yes	Yes	No
P-glycoprotein I inhibitor	Yes	Yes	No
P-glycoprotein II inhibitor	Yes	Yes	No
*Distribution*			
VDss (human) (log L/kg)	− 0.834	− 0.818	− 0.546
Fraction unbound (human) (Fu)	0.007	0.001	0.918
BBB permeability (log BB)	− 0.83	− 0.816	− 0.868
CNS permeability (log PS)	− 2.523	− 2.523	− 4.445
*Metabolism*			
CYP2D6 substrate	No	No	No
CYP3A4 substrate	Yes	Yes	No
CYP1A2 inhibitior	No	No	No
CYP2C19 inhibitior	No	No	No
CYP2C9 inhibitior	No	No	No
CYP2D6 inhibitior	No	No	No
CYP3A4 inhibitior	No	No	No
*Excretion*			
Total clearance (log ml/min/kg)	1.159	1.144	0.364
Renal OCT2 substrate	No	No	No
*Toxicity*			
AMES toxicity	No	No	No
Max. tolerated dose (human) (log mg/kg/day)	− 1.547	− 1.552	1.27
hERG I inhibitor	No	No	No
hERG II inhibitor	No	No	No
Oral rat acute toxicity (LD50) (mol/kg)	3.555	3.664	1.861
Oral rat chronic toxicity (LOAEL) (log mg/kg_bw/day)	0.727	0.544	2.605
Hepatotoxicity	No	No	No
Skin sensitization	No	No	No
*T. Pyriformis* toxicity (log ug/L)	0.296	0.295	0.136
Minnow toxicity (log mM)	0.683	0.881	3.915

**Table 6:** Drug-likeness analysis of *6a*, *6b* and AAZ

Lipinski’s five criteria	Accepted range	*6a*		*6b*		*AAZ*	
		Value	Result	Value	Result	Value	Result
Molecular weight (Da) (MW)	≤ 500	550	✓	550.81	–	194.09	✓
Num. H-bond donors	≤ 5	3	✓	3	✓	1	✓
Num. H-bond acceptors	≤ 10	6	✓	6	✓	6	✓
LogP	≤ 5	3.94	✓	3.94	✓	− 2.36	✓

The drug-likeness properties of the most active compounds were examined according to Lipinski’s rule of five, and the results are presented in Table 6. According to Lipinski’s rule of five, chemical structure limitations are defined as ≤ 500 for molecular weights, ≤ 10 for hydrogen bond acceptor numbers, ≤ 5 for hydrogen bond donor numbers, and ≤ 5 for the lipophilicity (log P or clog P) of compounds. An orally active drug-like compound should not violate the above criteria more than once according to the Lipinski rule. The drug-likeness study revealed that compounds ***6a*** and ***6b*** did not have a problem according to the five criteria of Lipinski, although there was a violation in one of the criteria (MW).

### Molecular dynamics simulation results

MD simulations are a powerful tool to measure the time-dependent behaviour of molecular systems with biological activity and to theoretically explain macromolecular structure–function interactions. In this study, MD simulations were performed to evaluate the dynamic states and binding stability of complexes formed with ***6a*** and ***6b***, which are potential inhibitor candidates for hCA I and hCA II receptors.

#### RMSD

**Fig. 9:**
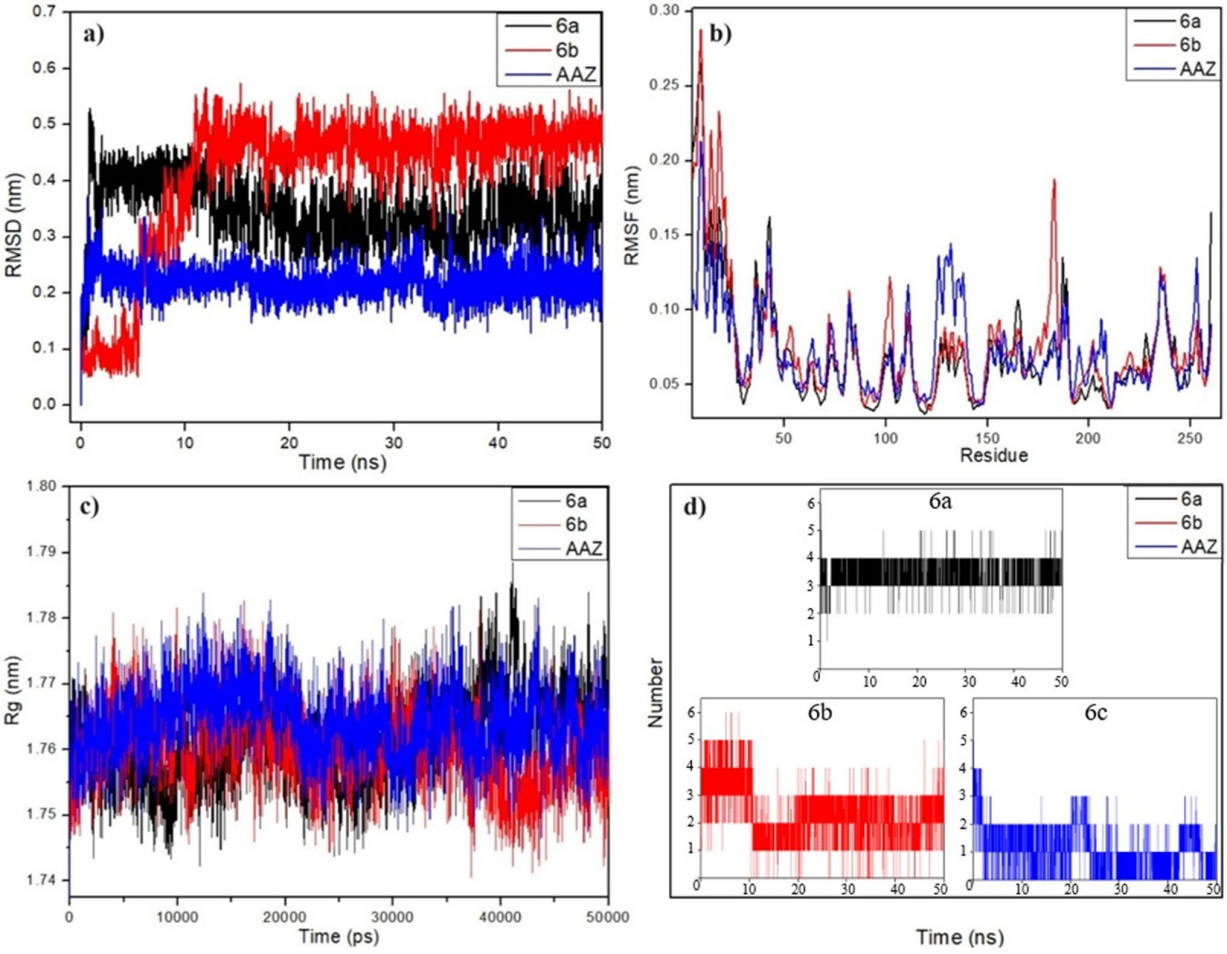
MD simulations of complexes between hCA I and ***6a***, ***6b*** and ***AAZ***. **a** root mean square deviation (RMSD), **b** root mean square fluctuation (RMSF), **c** radius of gyration (Rg) and **d** hydrogen bond number (H bond) of the complexes

RMSD analysis in molecular dynamics simulations provides important information about the stability and behaviour of systems by measuring the structural changes of a protein or protein–ligand complex over time. The calculated RMSD plots of the complexes of compounds ***6a***, ***6b*** and ***AAZ*** with hCA I and hCA II during a 50 ns simulation are shown in Fig. 9a, b, respectively.

The RMSD plot of the hCA I@***6a*** complex, represented in black in Fig. 9a, showed 0.5 nm at the beginning of the simulation, while it decreased over time and exhibited a stable movement around 0.31 nm until the end of the simulation after 12 ns. On the other hand, the RMSD curve of hCA I@***6b***, represented by the red colour, moved around 0.1 nm until 7 ns and then increased with time until 12 ns and stabilised around 0.46 nm until the end of the simulation. The RMSD plot of hCA I@***AAZ***, represented in blue colour, increased up to 0.38 nm at the beginning of the simulation and decreased again and stabilised around 0.21 nm in the range of 3–50 ns until the end of the simulation around 0.21 nm.

**Fig. 10:**
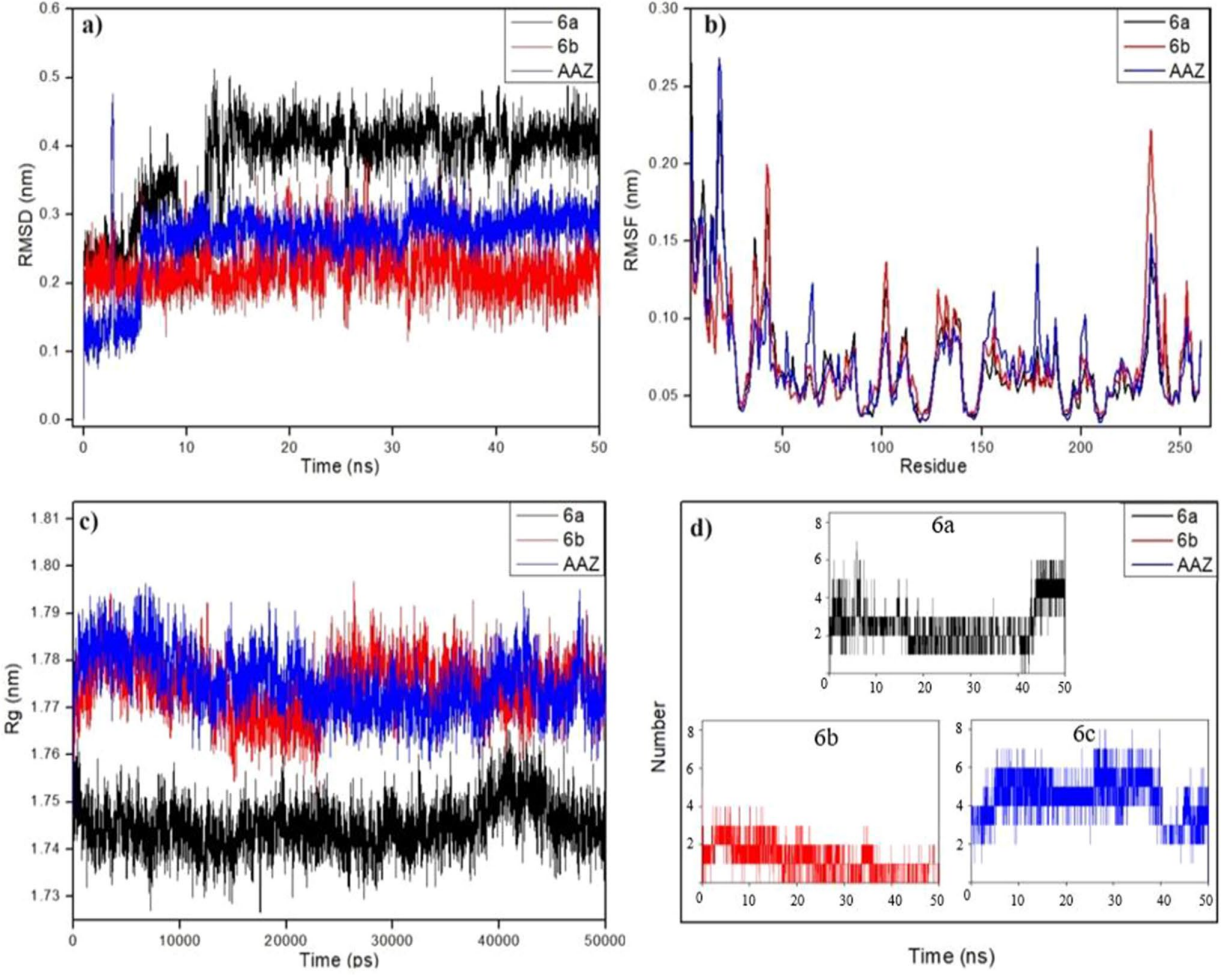
MD simulations of complexes between hCA II and **6a**, **6b** and **AAZ**. **a** root mean square deviation (RMSD), **b** root mean square fluctuation (RMSF), **c** radius of gyration (Rg) and **d** hydrogen bond number (H-bond) of the complexes

Similarly, when the RMSD plots of the hCA II complexes given in Fig. 10a are examined, the RMSD plot of the hCA II@***6a*** complex (black) moved between 0.15 and 0.51 nm in the 0–13 ns period, stabilised around 0.41 nm after 13 ns and moved stably until the end of the simulation. The RMSD curve (red) of hCA II@***6b*** stabilised around 0.20 nm at the beginning of the simulation and remained stable until the end of the simulation. The RMSD (blue) plot of hCA II@***AAZ*** gradually increased in the period of 0–6 ns, stabilized around 0.27 nm, and moved stably until the end of the simulation.

#### RMSF

RMSF plots provide information on the flexibility and mobility of complexes by providing information on the average atomic structure and fluctuations in protein residues during simulation. In this study, RMSF analysis was performed to analyse the flexibility and mobility of the complexes of compounds ***6a***, ***6b*** and ***AAZ*** with hCA I and hCA II and are given in Figs. 9b and 10b, respectively. The RMSF plots in Figs. 9b and 10b showed that the residues contributing to the fluctuations were relatively similar. Furthermore, the RMSF plots also showed that the average RMSF values of hCA I@***6a***, hCA I@***6b***, hCA I@***AAZ***, hCA II@***6a***, hCA II@***6b*** and hCA II@***AAZ*** complexes were 0.071, 0.077, 0.073, 0.073, 0.073, 0.073, 0.072, 0.073 and 0.072, respectively. The fact that the residue-induced fluctuations and RMSF averages in the complexes were quite similar to the reference compound ***AAZ*** indicated that ***6a*** and ***6b*** were well located to the binding site of hCA I and hCA II receptors.

### Radius of gyration (Rg)

In complexes, small Rg values represent a tight packing and compactness of the complex, while high Rg values represent a loose packing and reduced compactness [45]. The compactness of the complexes was analyzed through the gyration radii Rg drawings prepared using the “gyrate” tool. The plots obtained from the analysis of the compactness of hCA I with ***6a***, ***6b*** and ***AAZ*** and hCA II with ***6a***, ***6b*** and ***AAZ*** are shown in Figs. 9c and 10c, respectively. The hCA I complexes showed stable Rg values with insignificant differences around 1.76–1.77 nm from the beginning of the simulation as seen in Fig. 9c. The average Rg values for hCA I@***6a***, hCA I@***6b*** and hCA I@***AAZ*** were 1.762, 1.761 and 1.764 nm, respectively. These values indicate that hCA I complexes have approximately similar compactness. Similarly, as seen in Fig. 10c, the Rg values of hCA II@***6b*** and hCA II@***AAZ*** complexes are stable and insignificantly different in the range of 1.77–1.78 nm, respectively. However, when attention is paid to Fig. 10c, the Rg value of hCA II@***6a*** complex is around 1.75 nm, different from ***6b*** and ***AAZ***. These values show that ***6b*** and ***AAZ*** have similar compactness while ***6a*** is more compact than them in hCA II complexes.

### H-bond analysis

In complexes, H-bonds are one of the most important forces that ensure the interaction between ligand and protein and are responsible for the stability of the complexes. To interpret the binding affinity of compounds ***6a***, ***6b*** and ***AAZ*** with hCA I and hCA II proteins, respectively, the total number of bonds formed during protein–ligand interactions was calculated by H-bond analysis performed using the MD trajectories and shown in Fig. 9d and 10d. The H-bond analysis showed that the number of hydrogen bonds formed in the hCA I@***6a*** and hCA I@***6b*** complexes was between 1–5 and 0–6, respectively, while this value was between 0 and 4 in the hCA I@***AAZ*** complex formed by the reference compound ***AAZ***. H-bond analysis also showed that the number of hydrogen bond numbers in the hCA II@***6a***, hCA II@***6b*** and hCA II@***AAZ*** complexes was between 1–7, 0–4 and 1–8, respectively. Comparison of the results of H-bond analysis with ***AAZ*** showed a significant number of hydrogen bonds in the complexes hCA I@***6a***, hCA I@***6b***, hCA II@***6a*** and hCA II@***6b***, indicating a large number of interactions between proteins and ligands. The results also showed that the hydrogen bonds formed in the complexes were continuous during the simulations for each of the complexes and contributed significantly to the stability.

## Conclusion

Considering the pharmacological importance of pyrazoles due to their drug-like properties and the metabolic importance as CA inhibitors, the inhibition effects of the newly synthesized pyrazole-carboxamides (***6a*–*i***) on hCA I and hCA II isoenzymes were investigated. According to the inhibition data some of the synthesized molecules (***6a*** and*** 6b***) were evaluated as potent inhibitors for hCAs. It is promising that synthesized molecules were selective for hCA II. Besides, modifying the tails of compounds can contribute to increasing isoform selectivity. Compound ***6a*** showed the most potent inhibitory effect for hCA I and hCA II among the tested compounds. So this compound with a *K*_i_ value of 7 nM for hCA II can be considered as a potential candidate for further studies. The molecular docking results were confirmed by MD simulations. MD simulations were performed for 50 ns for the complexes formed between compounds ***6a***, ***6b*** and ***AAZ*** and hCA I and hCA II receptors. The results of RMSD, RMSF, Rg and H-bond analysis performed on the MD simulation trajectories showed that the binding of the compounds to the enzyme in the complexes does not cause much instability, but on the contrary, most of the time, it stabilizes the structure. To sum up, in silico studies conducted between the most potent inhibitors and receptors showed better results than the ***AAZ*** revealing that the newly synthesized ***6a*** and ***6b*** compounds are potential inhibitor candidates for hCA I and hCA II enzymes.

## Experimental protocols

### General

Chemical compounds and solvents that were used in this study were purchased from different suppliers, this includes Merck, Sigma, Sigma-Aldrich, Fluka and Alfa-Aesar. Also the solvents were freshly distilled before use. Follow up of the reactions and checking the homogeneity of the compounds was made by TLC on DC-Alufolien 20 × 20 cm Kieselgel 60F 254 plates (Merck) and the spots were visualized by Camag TLC devices (Camag, Upland, CA, USA) UV (254 and 366 nm). The melting points were measured in open capillary tube method on Barnstead Electrothermal 9200 melting point apparatus (Electrothermal Co, Essex, UK) and uncorrected. Solvent were evaporated under vacuum using Heidolph Laborota 4003 rotary evaporator (Heidolph Instruments GmbH & Co. KG, Schwabach, Germany). IR spectra were recorded on Bruker Optics Vertex 70 branded device using KBr pellets in the range of 400‒4000 cm^−1^ (Bruker Optik GmbH, Ettlingen, Germany). All the compounds were dissolved in DMSO-*d*_6_ for NMR measurements. Carbon and proton NMR spectra were recorded on BRUKER Avance III spectrometer operating at 100 MHz (^13^C) and 400 MHz (^1^H) (Bruker Optics Inc., MA, USA). HRMS spectra were recorded on an Agilent 6530 Accurate-Mass(Q-TOF) LC–MS instrument (Agilent Tech., Santa Clara, CA).

### General procedure for synthesis of pyrazolo-3-carboxamide derivatives (*6a*–*i*)

4-benzoyl-1,5-diphenyl-1*H*-pyrazole-3-carbonyl chloride (***4***) (1 mmol) was dissolved in freshly distilled THF (25–30 ml). Then 2 mmol of appropriate sulfonamide derivative (***5a*–*i***) was added to this solution. The mixture was heated under reflux for 5 h. The solvent was evaporated in vacuo and the residue was washed with water. The crude product was filtered and recrystallized from appropriate solvent or solvent mixture.

### 4-benzoyl-1,5-diphenyl-N-(4-sulfamoylphenyl)-1H-pyrazole-3-carboxamide (*6a)*

Compound ***6a*** was synthesized from ***4*** (0.386 g, 1 mmol) and 4-aminobenzenesulfonamide (***5a***) (0.348 g, 2 mmol) according to the general procedure and the residue recrystallized from *n*-propanol to give white crystals. Yield 480 mg, 92%; mp: 263–265 °C; FT-IR (KBr, ν, cm^−1^): 3347 and 3257 (NH), 3063 (CH, aromatic), 1688 (C = O, ketone), 1658 (C = O, amide), 1593–1431 (C = C and C = N), 1320 and 1154 (S = O asym. and sym.); ^1^H NMR (400 MHz, DMSO-*d*_6_) δ_H_ (ppm): 10.78 (br, s, 1H, CONH), 7.88–7.18 (m, 19H, ArH), 7.44 (s, 2H, SO_2_NH_2_); ^13^C NMR (100 MHz, DMSO-*d*_6_) δ_C_ (ppm): 191.10 (C = O, ketone), 159.96 (C = O, amide), 145.71 (pyrazole C3), 144.02 (pyrazole C5), 120.33 (pyrazole C4), 141.80, 139.39, 138.99, 138.02, 133.85, 130.09, 129.80, 129.61, 129.50, 129.43, 129.06, 128.98, 128.17, 126.93, 126.65, 122.31; HRMS (ESI) m/z: [M + Na]^+^ calcd for C_29_H_22_N_4_O_4_SNa: 545.1259; found: 545.1251.

### 4-benzoyl-1,5-diphenyl-N-(3-sulfamoylphenyl)-1H-pyrazole-3-carboxamide (*6b*)

Compound ***6b*** was synthesized from ***4*** (0.386 g, 1 mmol) and 3-aminobenzenesulfonamide (***5b***) (0.355 g, 2 mmol) according to the general procedure and the residue recrystallized from ethanol to give bright white prisms. Yield 454 mg, 87%; mp: 146–148 °C; FT-IR (KBr, ν, cm^−1^): 3375 and 3295 (NH), 3071 (CH, aromatic), 1692 (C = O, ketone), 1657 (C = O, amide), 1597–1428 (C = C and C = N), 1321 and 1155 (S = O asym. and sym.); ^1^H NMR (400 MHz, DMSO-*d*_6_) *δ*_H_ (ppm): 10.83 (br, s, 1H, CONH), 8.37 (s, 1H, sulfamoylphenyl C2-H, ArH), 7.85–7.18 (m, 18H, ArH), 7.37 (s, 2H, SO_2_NH_2_); ^13^C NMR (100 MHz, DMSO-*d*_6_) *δ*_C_ (ppm): 191.17 (C = O, ketone), 159.89 (C = O, amide), 145.72 (pyrazole C3), 145.02 (pyrazole C5), 143.89 (sulfamoylphenyl C3), 121.42 (pyrazole C4), 117.71 (sulfamoylphenyl C2), 139.29, 139.03, 138.02, 133.87, 130.09, 129.78, 129.73, 129.61, 129.52, 129.38, 129.08, 128.98, 128.22, 126.58, 123.67, 122.33; HRMS (ESI) m/z: [M + Na]^+^ calcd for C_29_H_22_N_4_O_4_SNa: 545.1259; found: 545.1231.

### 4-benzoyl-1,5-diphenyl-N-(2-sulfamoylphenyl)-1H-pyrazole-3-carboxamide (*6c*)

Compound ***6c*** was synthesized from ***4*** (0.386 g, 1 mmol) and 2-aminobenzenesulfonamide (***5c***) (0.351 g, 2 mmol) according to the general procedure and the residue recrystallized from ethanol to give white crystals. Yield 418 mg, 80%; mp: 233–235 °C; FT-IR (KBr, ν, cm^−1^): 3419 and 3305 (NH), 3065 (CH, aromatic), 1701 (C = O, ketone), 1656 (C = O, amide), 1584–1433 (C = C and C = N), 1339 and 1155 (S = O asym. and sym.); ^1^H NMR (400 MHz, DMSO-*d*_6_) *δ*_H_ (ppm): 10.92 (s, 1H, CONH), 8.27 (d, *J* = 8.3 Hz, 1H, sulfamoylphenyl C6-H, ArH), 7.89–7.18 (m, 15H, ArH), 7.79 (br, s, 2H, SO_2_NH_2_), 7.60 (t, *J* = 7.4 Hz, 1H, sulfamoylphenyl C5-H, ArH), 6.82 (d, *J* = 8.2 Hz, 1H, sulfamoylphenyl C3-H, ArH), 6.62 (t, *J* = 8.1 Hz, 1H, sulfamoylphenyl C4-H, ArH); ^13^C NMR (100 MHz, DMSO-*d*_6_) *δ*_C_ (ppm): 191.23 (C = O, ketone), 159.00 (C = O, amide), 145.74 (pyrazole C3), 144.70 (pyrazole C5), 143.96 (sulfamoylphenyl C2), 122.18 (pyrazole C4), 138.96, 138.06, 135.03, 133.98, 133.44, 133.33, 131.96, 130.03, 129.92, 129.70, 129.60, 129.29, 129.15, 129.07, 128.47, 128.37, 128.19, 125.99, 124.92, 124.36, 122.61, 117.32, 115.66; HRMS (ESI) m/z: [M + Na]^+^ calcd for C_29_H_22_N_4_O_4_SNa: 545.1259; found: 545.1236.

### 4-benzoyl-1,5-diphenyl-N-(4-(N-(pyridin-2-yl)sulfamoyl)phenyl)-1H-pyrazole-3-carboxamide (*6d*)

Compound ***6d*** was synthesized from ***4*** (0.386 g, 1 mmol) and sulfapyridine (***5d***) (0.504 g, 2 mmol) according to the general procedure and the residue recrystallized from EtOH-DMF (9:1) to give off-white crystals. Yield 485 mg, 81%; mp: 239–241 °C; FT-IR (KBr, ν, cm^−1^): 3418 and 3242 (NH), 3056 (CH, aromatic), 1665 (C = O, ketone), 1636 (C = O, amide), 1594–1432 (C = C and C = N), 1393 and 1144 (S = O asym. and sym.); ^1^H NMR (400 MHz, DMSO-*d*_6_) *δ*_H_ (ppm): 11.88 (br, s, 1H, SO_2_NH), 10.82 (s, 1H, CONH), 8.01 (d, *J* = 4.2 Hz, 1H, pyridine C6-H), 7.96–7.17 (m, 19H, ArH), 7.55 (t, *J* = 7.3 Hz, 1H, pyridine C4-H), 7.13 (d, *J* = 8.6 Hz, 1H, pyridine C3-H), 6.86 (t, *J* = 6.2 Hz, 1H, pyridine C5-H); ^13^C NMR (100 MHz, DMSO-*d*_6_) *δ*_C_ (ppm): 191.08 (C = O, ketone), 162.78 (C = O, amide), 159.95 (pyridine C2), 153.45 (pyridine C6), 145.64 (pyrazole C3), 144.04 (pyrazole C5), 120.31 (pyrazole C4), 114.03 (pyridine C3), 142.68, 142.21, 138.97, 138.01, 133.82, 130.08, 129.78, 129.60, 129.54, 129.47, 129.42, 129.05, 128.96, 128.59, 128.16, 128.01, 126.65, 122.33; HRMS (ESI) m/z: [M + Na]^+^ calcd for C_34_H_25_N_5_O_4_SNa: 622.1525; found: 622.1527.

### 4-benzoyl-1,5-diphenyl-N-(4-(N-(pyrimidin-2-yl)sulfamoyl)phenyl)-1H-pyrazole-3-carboxamide (*6e*)

Compound ***6e*** was synthesized from ***4*** (0.386 g, 1 mmol) and sulfadiazine (***5e***) (0.521 g, 2 mmol) according to the general procedure and the residue recrystallized from ethanol to give cream-colored crystals. Yield 510 mg, 85%; mp: 244–246 °C; FT-IR (KBr, ν, cm^−1^): 3379 (NH), 3040 (CH, aromatic), 1690 (C = O, ketone), 1664 (C = O, amide), 1594–1440 (C = C and C = N), 1334 and 1167 (S = O asym. and sym.); ^1^H NMR (400 MHz, DMSO-*d*_6_) *δ*_H_ (ppm): 11.69 (br, s, 1H, SO_2_NH), 10.88 (s, 1H, CONH), 8.50 (d, *J* = 4.8 Hz, 2H, pyrimidine C4-H and C6-H), 7.89–7.17 (m, 19H, ArH), 7.04 (t, *J* = 4.7 Hz, 1H, pyrimidine C5-H); ^13^C NMR (100 MHz, DMSO-*d*_6_) *δ*_C_ (ppm): 191.07 (C = O, ketone), 160.02 (C = O, amide), 158.82 (pyrimidine C2), 157.38 (pyrimidine C4 and C6), 145.58 (pyrazole C3), 144.04 (pyrazole C5), 120.12 (pyrazole C4), 116.28 (pyrimidine C5), 142.81, 138.96, 138.00, 135.20, 133.84, 130.29, 130.08, 129.79, 129.60, 129.48, 129.44, 129.06, 128.97, 128.14, 126.65, 122.36; HRMS (ESI) m/z: [M + Na]^+^ calcd for C_33_H_24_N_6_O_4_SNa: 623.1477; found: 623.1504.

### 4-benzoyl-N-(4-(N-(4-methylpyrimidin-2-yl)sulfamoyl)phenyl)-1,5-diphenyl-1H-pyrazole-3-carboxamide (*6f*)

Compound ***6f*** was synthesized from ***4*** (0.386 g, 1 mmol) and sulfamerazine (***5f***) (0.539 g, 2 mmol) according to the general procedure and the residue recrystallized from EtOH-DMF (3:1) to give white crystals. Yield 528 mg, 86%; mp: 237–239 °C; FT-IR (KBr, ν, cm^−1^): 3415 (NH), 3062 (CH, aromatic), 2924 (CH, aliphatic), 1689 (C = O, ketone), 1664 (C = O, amide), 1595–1424 (C = C and C = N), 1336 and 1163 (S = O asym. and sym.); ^1^H NMR (400 MHz, DMSO-*d*_6_) *δ*_H_ (ppm): 11.63 (br, s, 1H, SO_2_NH), 10.86 (s, 1H, CONH), 8.31 (d, *J* = 5.1 Hz, 1H, pyrimidine C6-H), 7.92–7.16 (m, 19H, ArH), 6.90 (d, *J* = 5.1 Hz, 1H, pyrimidine C5-H), 2.30 (s, 3H, CH_3_); ^13^C NMR (100 MHz, DMSO-*d*_6_) *δ*_C_ (ppm): 191.08 (C = O, ketone), 169.38 (pyrimidine C2), 162.79 (pyrimidine C4), 160.00 (C = O, amide), 157.01 (pyrimidine C6), 145.59 (pyrazole C3), 144.02 (pyrazole C5), 119.95 (pyrazole C4), 100.00 (pyrimidine C5), 23.75 (CH_3_), 142.68, 138.97, 137.99, 133.84, 130.08, 129.79, 129.60, 129.49, 129.42, 129.26, 129.06, 129.01, 128.97, 128.15, 126.63, 122.35; HRMS (ESI) m/z: [M + Na]^+^ calcd for C_34_H_26_N_6_O_4_SNa: 637.1634; found: 637.1618.

### 4-benzoyl-N-(4-(N-(3,4-dimethylisoxazol-5-yl)sulfamoyl)phenyl)-1,5-diphenyl-1H-pyrazole-3-carboxamide (*6 g*)

Compound ***6 g*** was synthesized from ***4*** (0.386 g, 1 mmol) and sulfisoxazole (***5 g***) (0.540 g, 2 mmol) according to the general procedure and the residue recrystallized from methanol to give bright cream-colored flakes. Yield 543 mg, 88%; mp: 165–167 °C; FT-IR (KBr, ν, cm^−1^): 3354 and 3224 (NH), 3061 (CH, aromatic), 2960 (CH, aliphatic), 1686 (C = O, ketone), 1666 (C = O, amide), 1593–1428 (C = C and C = N), 1340 and 1160 (S = O asym. and sym.); ^1^H NMR (400 MHz, DMSO-*d*_6_) *δ*_H_ (ppm): 10.95 (s, 2H, 2xNH, SO_2_NH submerged under CONH), 7.96–7.19 (m, 19H, ArH), 2.08 (s, 3H, isoxazole-CH_3_(3)), 1.63 (s, 3H, isoxazole-CH_3_(4)); ^13^C NMR (100 MHz, DMSO-*d*_6_) *δ*_C_ (ppm): 191.07 (C = O, ketone), 161.89 (isoxazole C3), 160.11 (C = O, amide), 155.99 (isoxazole C5), 145.56 (pyrazole C3), 144.08 (pyrazole C5), 120.56 (pyrazole C4), 105.58 (isoxazole C4), 10.81 (isoxazole-CH_3_(3)), 6.35 (isoxazole-CH_3_(4)), 143.24, 138.97, 138.00, 134.82, 134.75, 133.86, 130.10, 129.80, 129.61, 129.50, 129.45, 129.07, 128.97, 128.14, 126.65, 122.39; HRMS (ESI) m/z: [M + Na]^+^ calcd for C_34_H_27_N_5_O_5_SNa: 640.1631; found: 640.1611.

### 4-benzoyl-1,5-diphenyl-N-(4-(N-(thiazol-2-yl)sulfamoyl)phenyl)-1H-pyrazole-3-carboxamide (*6h*)

Compound ***6h*** was synthesized from ***4*** (0.386 g, 1 mmol) and sulfathiazole (***5h***) (0.521 g, 2 mmol) according to the general procedure and the residue recrystallized from EtOH-DMF (8:1) to give bright white crystals. Yield 538 mg, 89%; mp: 252–254 °C; FT-IR (KBr, ν, cm^−1^): 3415 and 3372 (NH), 3059 (CH, aromatic), 1679 (C = O), 1592–1424 (C = C and C = N), 1330 and 1145 (S = O asym. and sym.); ^1^H NMR (400 MHz, DMSO-*d*_6_) *δ*_H_ (ppm): 12.71 (br, s, 1H, SO_2_NH), 10.82 (s, 1H, CONH), 7.86–7.24 (m, 19H, ArH), 7.18 (d, *J* = 6.9 Hz, 1H, thiazole C4-H), 6.81 (d, *J* = 4.5 Hz, 1H, thiazole C5-H); ^13^C NMR (100 MHz, DMSO-*d*_6_) *δ*_C_ (ppm): 191.10 (C = O, ketone), 169.23 (thiazole C2), 159.92 (C = O, amide), 145.68 (pyrazole C3), 144.01 (pyrazole C5), 137.44 (thiazole C4), 120.34 (pyrazole C4), 108.59 (thiazole C5), 142.06, 138.98, 138.02, 133.83, 130.09, 129.78, 129.60, 129.48, 129.42, 129.05, 128.97, 128.17, 127.17, 126.65, 124.87, 122.32; HRMS (ESI) m/z: [M + Na]^+^ calcd for C_32_H_23_N_5_O_4_S_2_Na: 628.1089; found: 628.1065.

### 4-benzoyl-N-(4-(N-carbamimidoylsulfamoyl)phenyl)-1,5-diphenyl-1H-pyrazole-3-carboxamide (*6i*)

Compound ***6i*** was synthesized from ***4*** (0.386 g, 1 mmol) and sulfaguanidine (***5i***) (0.433 g, 2 mmol) according to the general procedure and the residue recrystallized from EtOH-DMF (5:1) to give bright straw-colored needles. Yield 468 mg, 83%; mp: 280–282 °C; FT-IR (KBr, ν, cm^−1^): 3427 and 3323 (NH), 3063 (CH, aromatic), 1669 (C = O, ketone), 1642 (C = O, amide), 1594–1429 (C = C and C = N), 1397 and 1144 (S = O asym. and sym.); ^1^H NMR (400 MHz, DMSO-*d*_6_) *δ*_H_ (ppm): 10.76 (s, 1H, CONH), 7.82–7.17 (m, 19H, ArH), 6.67 (br, s, 4H, 2xNH and NH_2_); ^13^C NMR (100 MHz, DMSO-*d*_6_) *δ*_C_ (ppm): 191.12 (C = O, ketone), 159.86 (C = O, amide), 158.52 (guanidine C = NH), 145.78 (pyrazole C3), 144.00 (pyrazole C5), 120.18 (pyrazole C4), 141.27, 139.89, 139.00, 138.04, 133.83, 130.09, 129.78, 129.61, 129.49, 129.41, 129.06, 128.97, 128.19, 126.83, 126.65, 122.29; HRMS (ESI) m/z: [M + Na]^+^ calcd for C_30_H_24_N_6_O_4_SNa: 587.1477; found: 587.1469.

### Purification of hCA I and hCA II

Purification of enzymes have described in detail in our previous studies [46–49]. In brief, blood samples collected in anticoagulated tubes have centrifuged, erythrocytes have separated and hemolyzed. Following centrifugation, the supernatant (pH 8.7) was loaded onto the Sepharose®-4B-L-tyrosine-*p*-aminobenzenesulfonamide column. After extensive washing, the hCA I and II isoenzymes were eluted with 1.0 M NaCl/25.0 mM Na_2_HPO_4_ (pH 6.3) and 0.1 M CH_3_COONa/0.5 M NaClO_4_ (pH 5.6) [50]. Protein quantity in eluates was determined [51]. The purified enzymes have characterized by SDS-PAGE analysis in the presence of protein marker [52].

### Esterase activity assay and determination of IC_50_ values and inhibition constants (K_i_)

Esterase activities of purified hCA I and hCA II were determined according to the literature [53]. Activity measurement has described in detail in our previous studies [46–49].

IC_50_ values were determined by measuring esterase activity in the presence of compounds (***6a*–*i***). Regression analysis graphs have drawn by plotting inhibitor concentrations vs. percent enzyme activity by using Microsoft Excel Package Program (Microsoft Office 2016).

The method for determination of *K*_i_ values has described in detail in our previous studies [46–49]. In brief, to determine *K*_i_ values, esterase activity measurements have performed at five different substrate concentrations for each of the three different inhibitor concentrations (30, 50 and 70% inhibition effect). The data have linearized with Lineweaver–Burk plot in order to obtain *K*_i_ values [54].

IC_50_ and *K*_i_ values were expressed by averaging the results from triplicate experiments.

### Molecular docking study

Among the newly synthesized compounds, which will be used as ligands in the molecular docking study, molecular structure of the most active ***6a*** and ***6b*** compounds were designed with the help of the Gaussview 6.0 molecular visualition program [55]. The most stable molecular structures of compounds ***6a*** and ***6b*** were calculated using the Gaussian 09 package software [56] DFT/B3LYP level and the 6-31G(d) basis set. X-ray crystal structures of hCA I and hCA II receptors (PDB code: 2CAB and 1CA2, respectively) to be used in the molecular docking process were taken from the RCSB Protein Data Bank in pdb format. The crystal structures of these downloaded receptors were used in molecular docking studies were performed with the hybrid use of UCSF Chimera software and AutoDock Vina tool [57]. Then, with the UCSF Chimera software for the molecular docking process, water molecules were removed from the receptor structures, hydrogen atoms were added, apolar hydrogen atoms were joined, and finally Kollman charges were added. Ligands were added to the structures and rotatable bonds were determined for the ligands. Finally, the docking studies of hCA I and hCA II receptors and ***6a*** and ***6b***, respectively, were performed to investigate the number, length and other interactions of hydrogen bonds formed between protein and ligands. The grid map was defined by UCSF Chimera software to include active site residues. Molecular docking studies were performed using the “blind insertion” method. Binding score was calculated using the Lamarckian Genetic Algorithm. Final conformations and docking scores of the complexes were determined according to the lowest negative binding score [58–60].

### ADMET and drug-likeness study

Pharmacological and drug-likeness properties of the most active compounds were determined by ADMET (Absorption, Distribution, Metabolism, Elimination and Toxicity) study [61]. Molecular structures of the compounds to be analyzed, previously prepared with the help of Gaussview 6 molecular visualition software, were converted to SMILES using OpenBabel software. Then, ADMET analysis was performed using the pkCSM online tool, which has a user-friendly and useful interface for the most active compounds [62]. Drug-likeness properties of the most active compounds were evaluated according to Lipinski’s five criteria.

### Molecular dynamic simulations

In order to observe the stability of hCA I@***6a***, hCA I@***6b***, hCA I@***AAZ***, hCA II@***6a***, hCA II@***6b*** and hCA II@***AAZ*** complexes obtained as a result of the molecular docking study, 50 ns MD simulations with a time step of 2 fs were performed using Gromacs 2023.3. The topologies of proteins were prepared using the pdb2gmx module integrated into GROMACS and the CHARMM36 all-atom force field [63]. Similarly, the topologies of ligands were prepared using the CHARMM force field with the open access SwisParam web server [64, 65]. Temperature control during the simulation was carried out with the help of Berendsen thermostat [66, 67]. Newton’s equations of motion are defined using the Leap-Frog algorithm and Berendsen weak coupling method, which are numerical integration algorithms for simulation [68]. For the energy minimisation of the complexes, a steepest descent minimisation algorithm with a maximum of 25.000 steps was applied. The resulting simulated trajectories were analysed for Root Mean Square Deviation (RMSD), Root Mean Square Fluctuation (RMSF), Radius of Gyration (Rg) and Hydrogen Bond (H-bond) using the GROMACS analysis tools.

## Supplementary Information

Below is the link to the electronic supplementary material.

Supplementary file1 (DOCX 1439 KB)
