# Supplementary material for: Synthesis, molecular docking and molecular dynamics simulations, drug-likeness studies, ADMET prediction and biological evaluation of novel pyrazole-carboxamides bearing sulfonamide moiety as potent carbonic anhydrase inhibitors
Source: Mol Divers. 2024 Jun 13;29(2):1207–27. doi: 10.1007/s11030-024-10901-0 (PMC11909088; doi:10.1007/s11030-024-10901-0)
Supplement: Supplementary file 1 — Supplementary file1 (DOCX 1439 KB) [file 11030_2024_10901_MOESM1_ESM.docx]

Supplementary Material

Contents

[^1^H NMR Data of compounds ***6a***-***i*** 3](#_Toc469829215)

[Figure S1: ^1^H NMR spectrum of compound ***6a*** 3](#_Toc469829216)

[Figure S2: ^1^H NMR spectrum of compound ***6b*** 3](#_Toc469829217)

[Figure S3: ^1^H NMR spectrum of compound ***6c*** 4](#_Toc469829218)

[Figure S4: ^1^H NMR spectrum of compound ***6d*** 4](#_Toc469829219)

[Figure S5: ^1^H NMR spectrum of compound ***6e*** 5](#_Toc469829220)

[Figure S6: ^1^H NMR spectrum of compound ***6f*** 5](#_Toc469829221)

[Figure S7: ^1^H NMR spectrum of compound ***6g*** 6](#_Toc469829222)

[Figure S8: ^1^H NMR spectrum of compound ***6h*** 6](#_Toc469829223)

[Figure S9: ^1^H NMR spectrum of compound ***6i*** 7](#_Toc469829224)

[^13^C NMR Data of compounds ***6a-i*** 7](#_Toc469829226)

[Figure S10: ^13^C NMR spectrum of compound ***6a*** 7](#_Toc469829233)

[Figure S11: ^13^C NMR spectrum of compound ***6b*** 8](#_Toc469829234)

[Figure S12: ^13^C NMR spectrum of compound ***6c*** 8](#_Toc469829235)

[Figure S13: ^13^C NMR spectrum of compound ***6d*** 9](#_Toc469829236)

[Figure S14: ^13^C NMR spectrum of compound ***6e*** 9](#_Toc469829222)

[Figure S15: ^13^C NMR spectrum of compound ***6f*** 10](#_Toc469829223)

[Figure S16: ^13^C NMR spectrum of compound ***6g*** 10](#_Toc469829224)

[Figure S17: ^13^C NMR spectrum of compound ***6h*** 11](#_Toc469829225)

[Figure S18: ^13^C NMR spectrum of compound ***6i*** 11](#_Toc469829227)

[Mass Spectra of compounds ***6a-i*** 12](#_Toc469829248)

[Figure S19: Mass spectra of compound ***6a*** 12](#_Toc469829238)

[Figure S20: Mass spectra of compound ***6b*** 13](#_Toc469829239)

[Figure S21: Mass spectra of compound ***6c*** 14](#_Toc469829240)

[Figure S22: Mass spectra of compound ***6d*** 15](#_Toc469829241)

[Figure S23: Mass spectra of compound ***6e*** 16](#_Toc469829242)

[Figure S24: Mass spectra of compound ***6f*** 17](#_Toc469829243)

[Figure S25: Mass spectra of compound ***6g*** 18](#_Toc469829244)

[Figure S26: Mass spectra of compound ***6h*** 19](#_Toc469829245)

[Figure S27: Mass spectra of compound ***6i*** 20](#_Toc469829246)

Purification of carbonic anhydrase I and II isoenzymes from human erythrocytes 21

Determination of hydratase and esterase activities of hCA I and hCA II 21

Determination of IC_50_ and *K*_i_ values of the compounds 22

Statistical analysis 22

**^1^H NMR Data of compounds *6a-i***


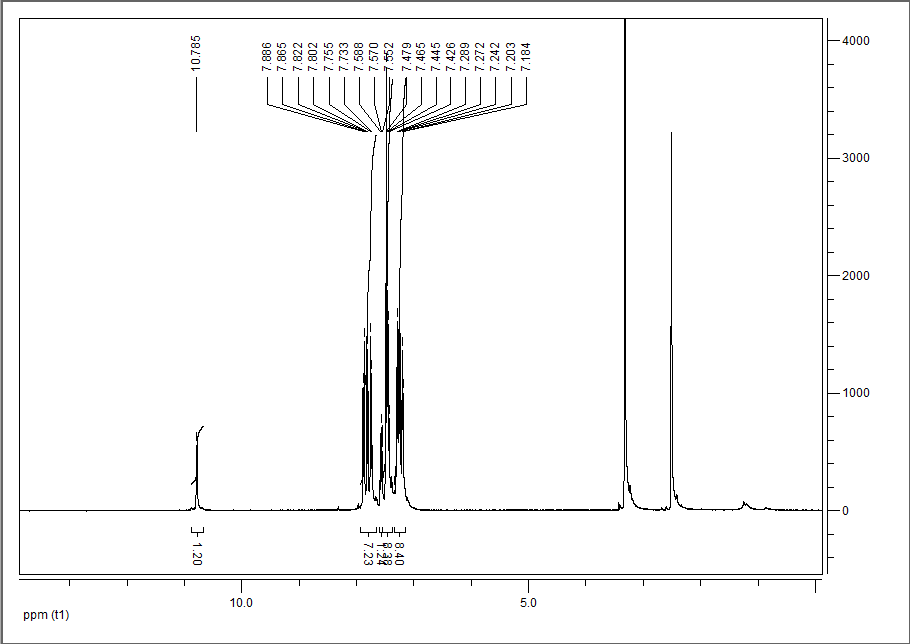


Figure S1: ^1^H NMR spectrum of compound ***6a***


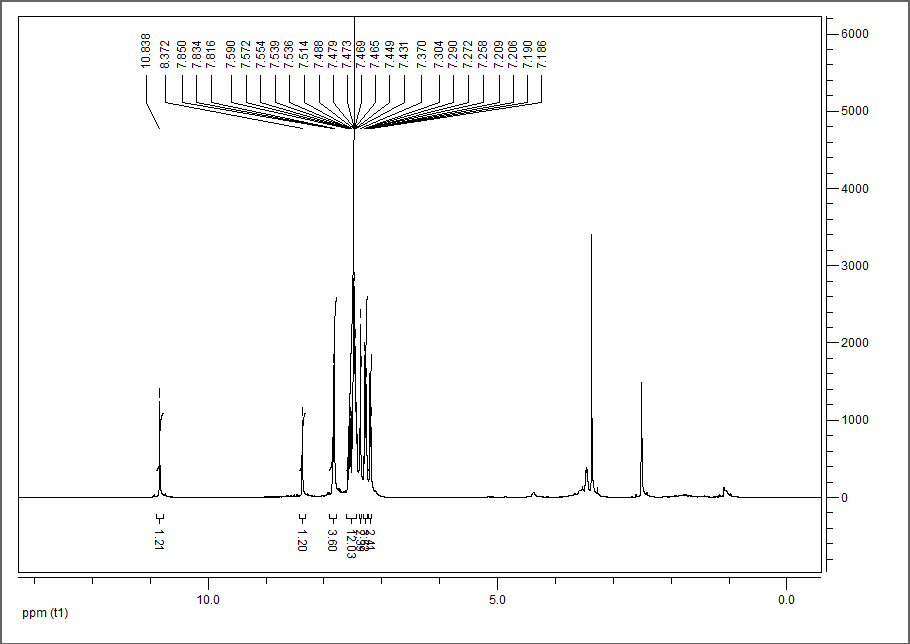


Figure S2: ^1^H NMR spectrum of compound ***6b***


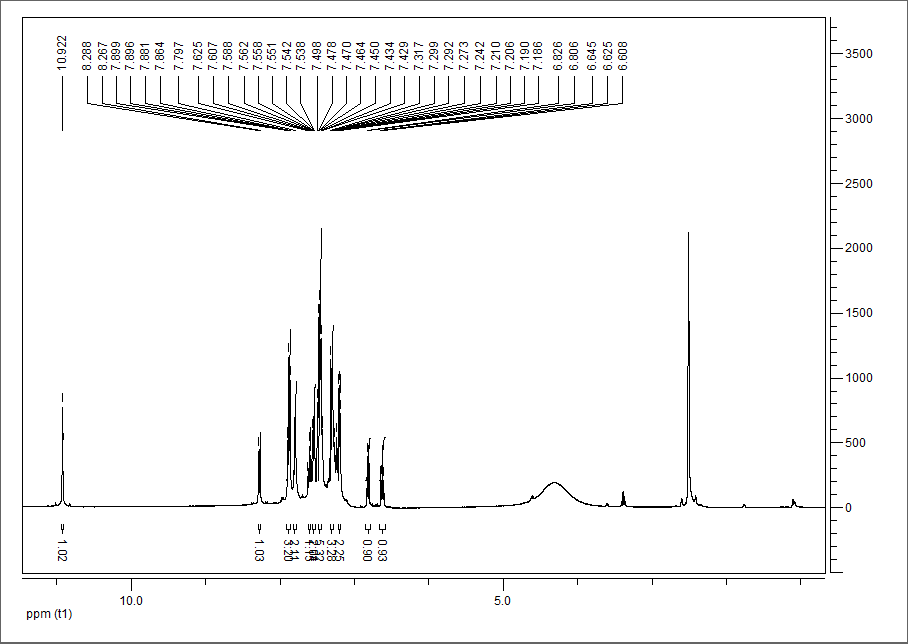


Figure S3: ^1^H NMR spectrum of compound ***6c***


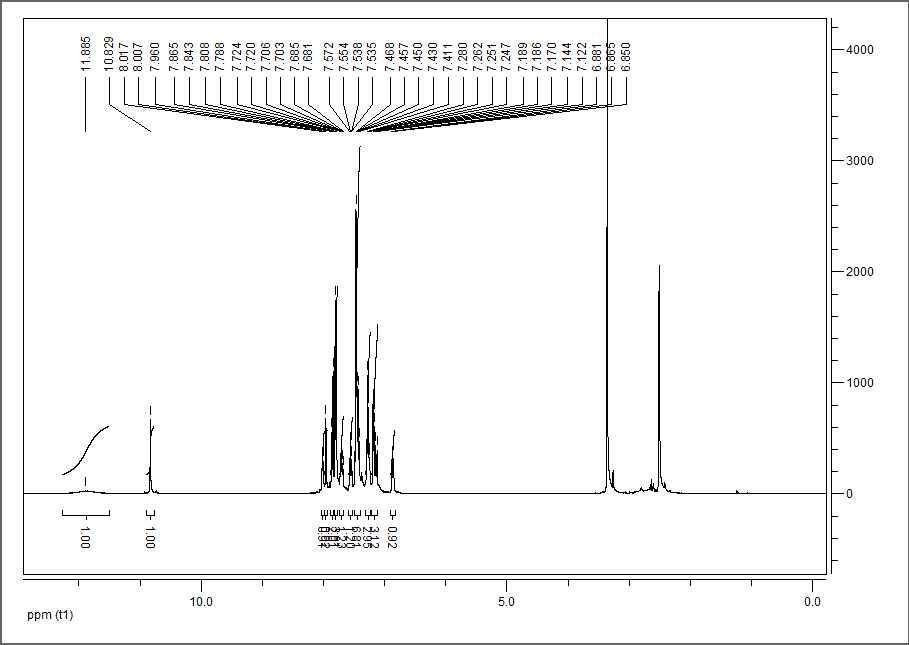


Figure S4: ^1^H NMR spectrum of compound ***6d***


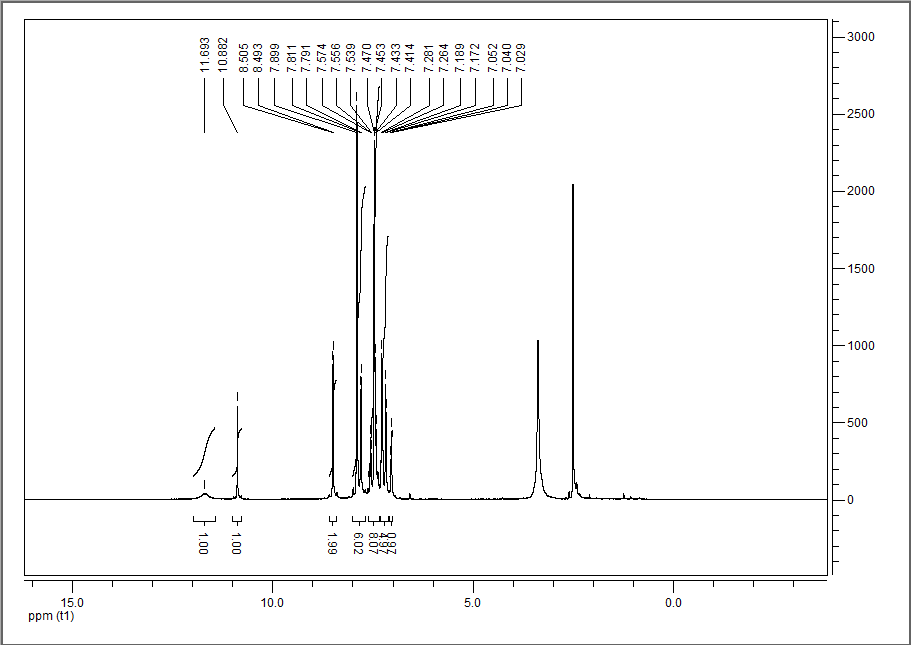


Figure S5: ^1^H NMR spectrum of compound ***6e***


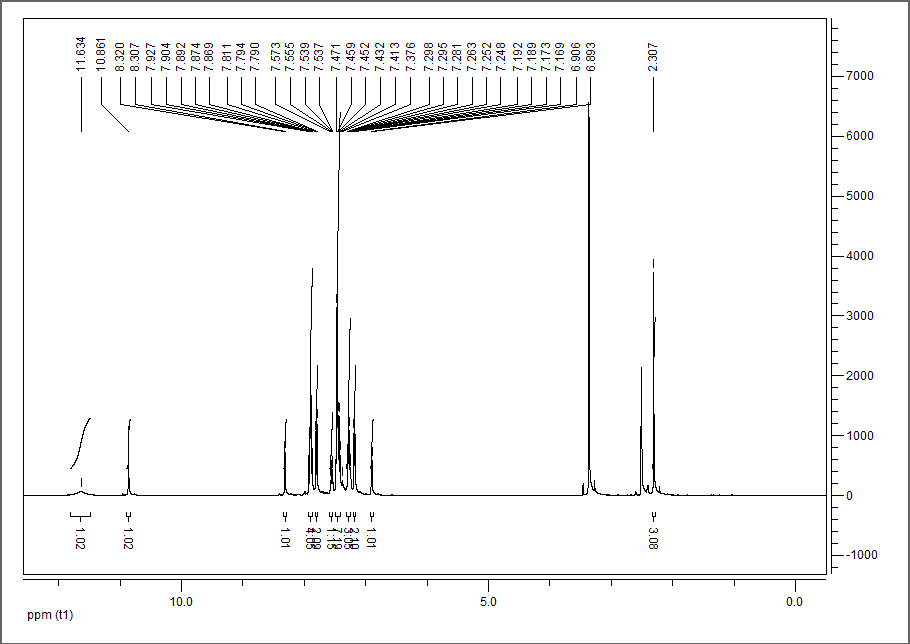


Figure S6: ^1^H NMR spectrum of compound ***6f***

Figure S7: ^1^H NMR spectrum of compound ***6g***


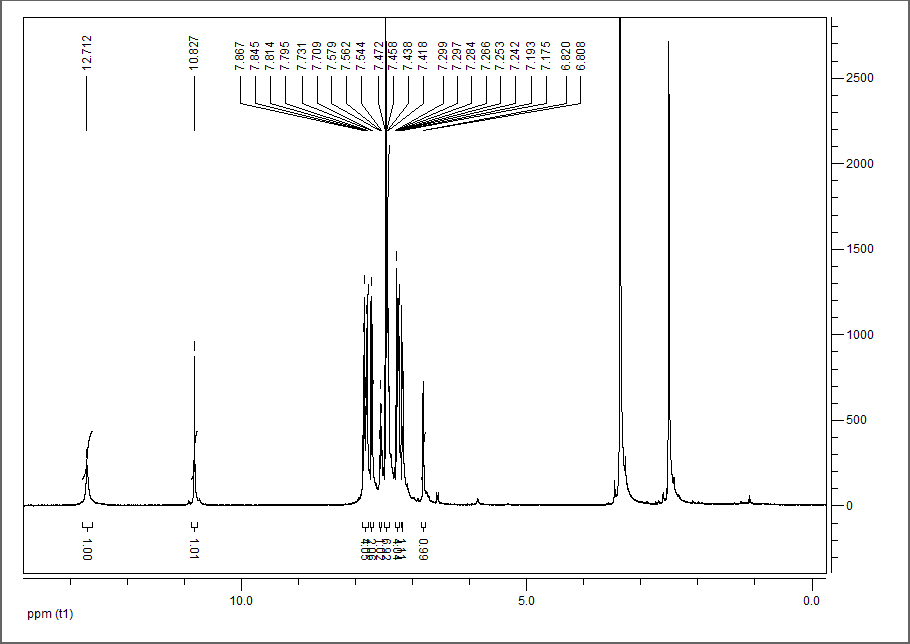


Figure S8: ^1^H NMR spectrum of compound ***6h***


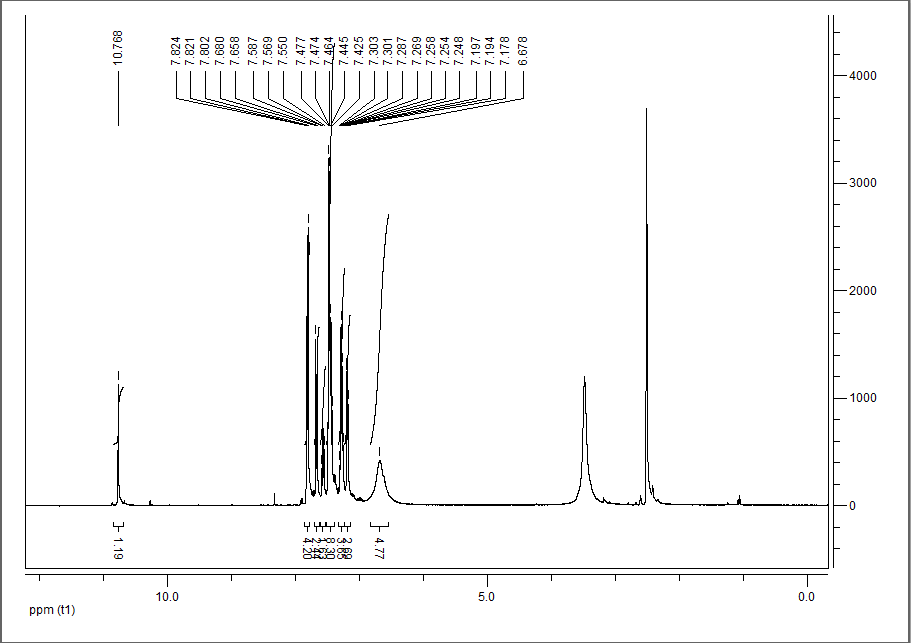


Figure S9: ^1^H NMR spectrum of compound ***6i***

**^13^C NMR Data of compounds *6a-i***


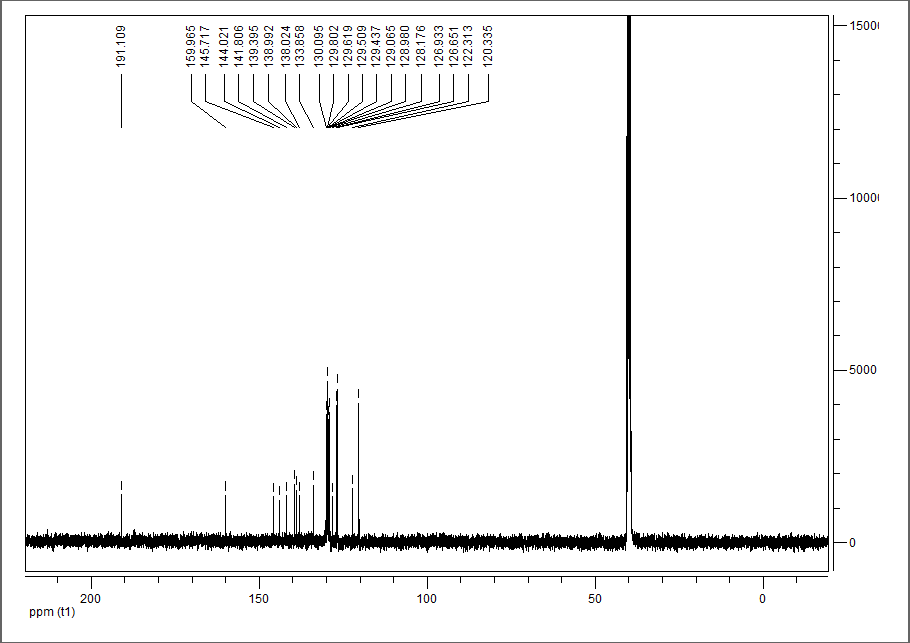


Figure S10: ^13^C NMR spectrum of compound ***6a***


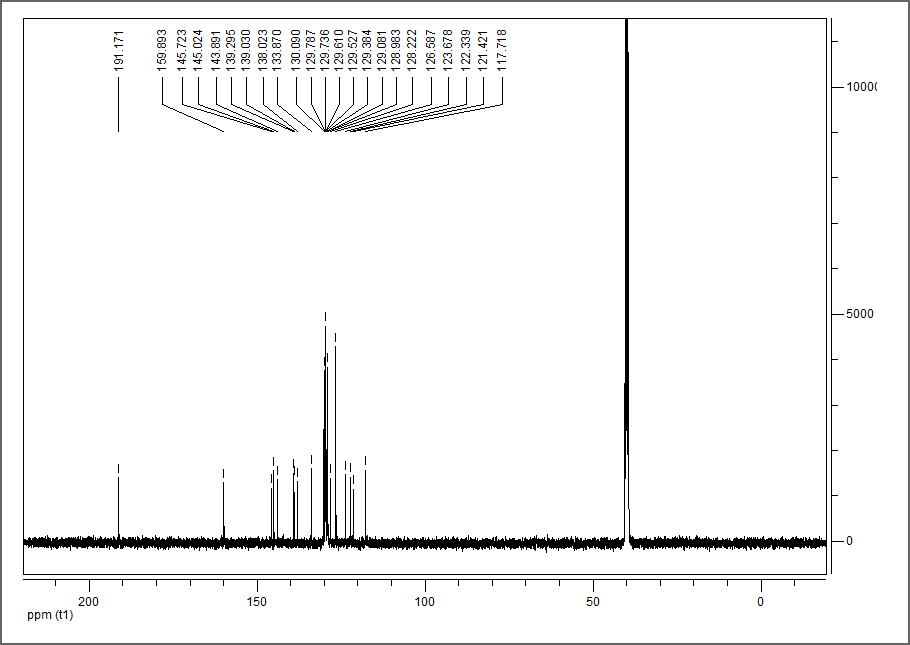


Figure S11: ^13^C NMR spectrum of compound ***6b***


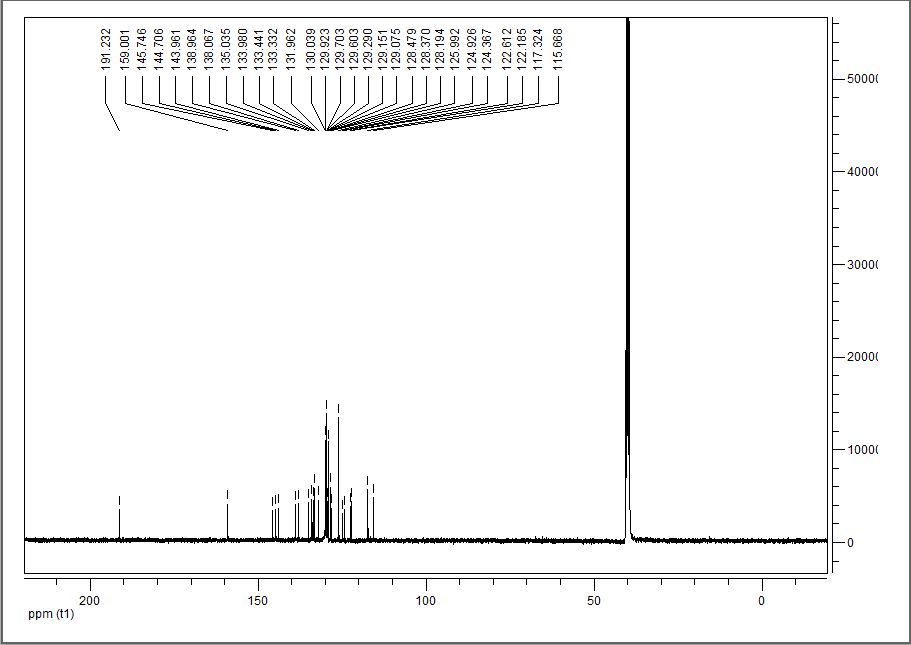


Figure S12: ^13^C NMR spectrum of compound ***6c***


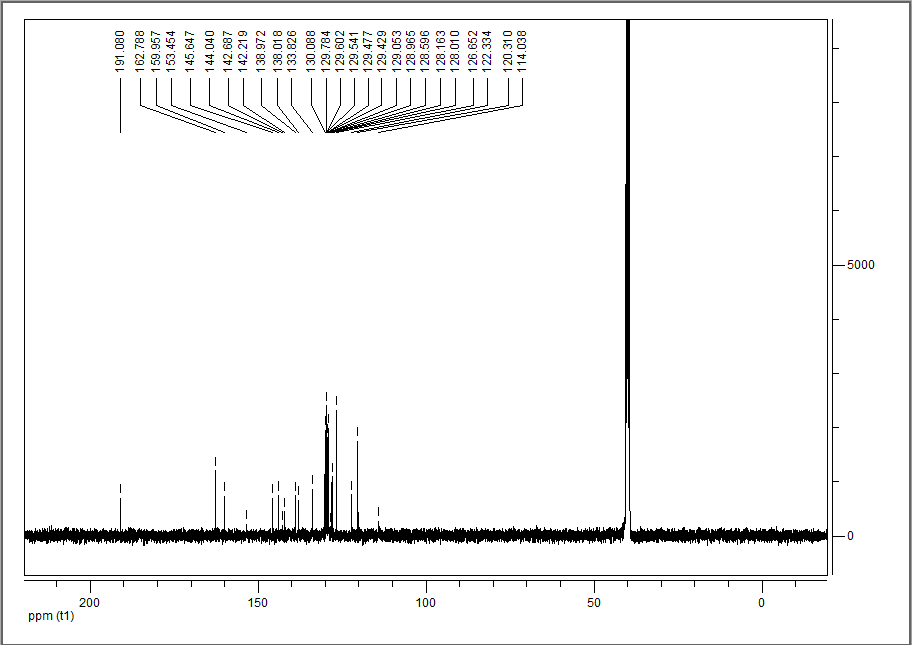


Figure S13: ^13^C NMR spectrum of compound ***6d***


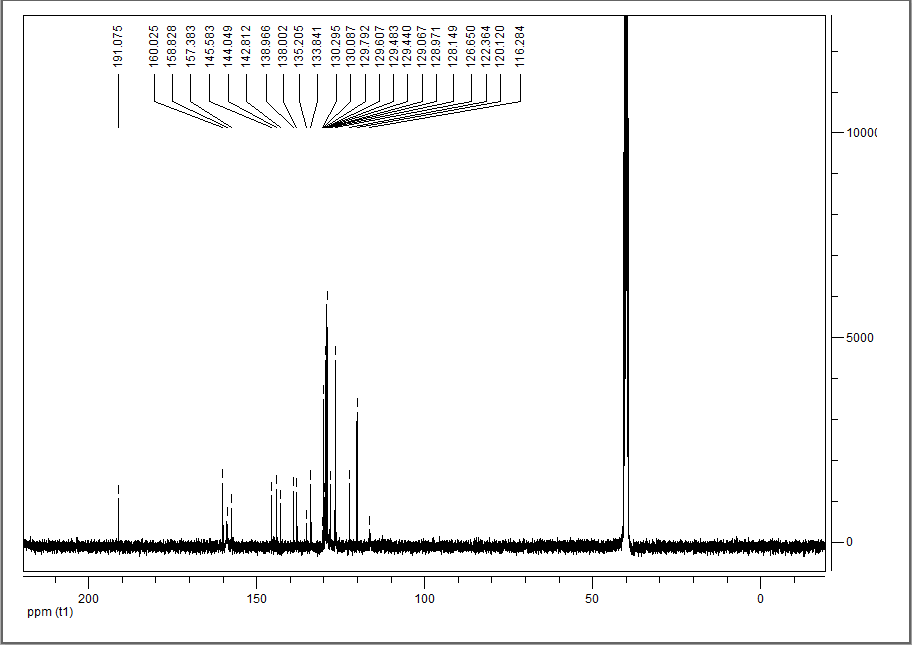


Figure S14: ^13^C NMR spectrum of compound ***6e***


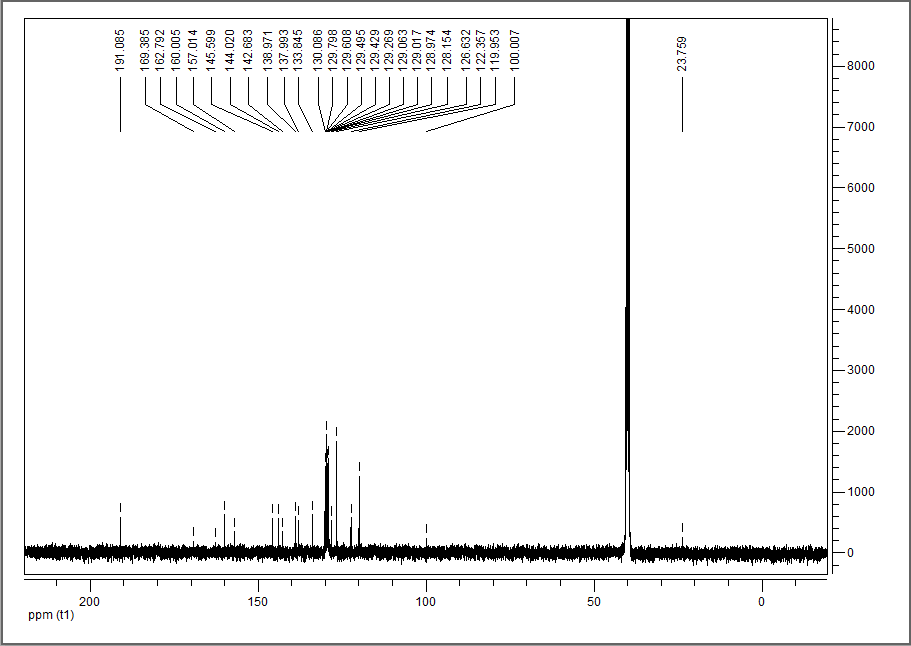


Figure S15: ^13^C NMR spectrum of compound ***6f***


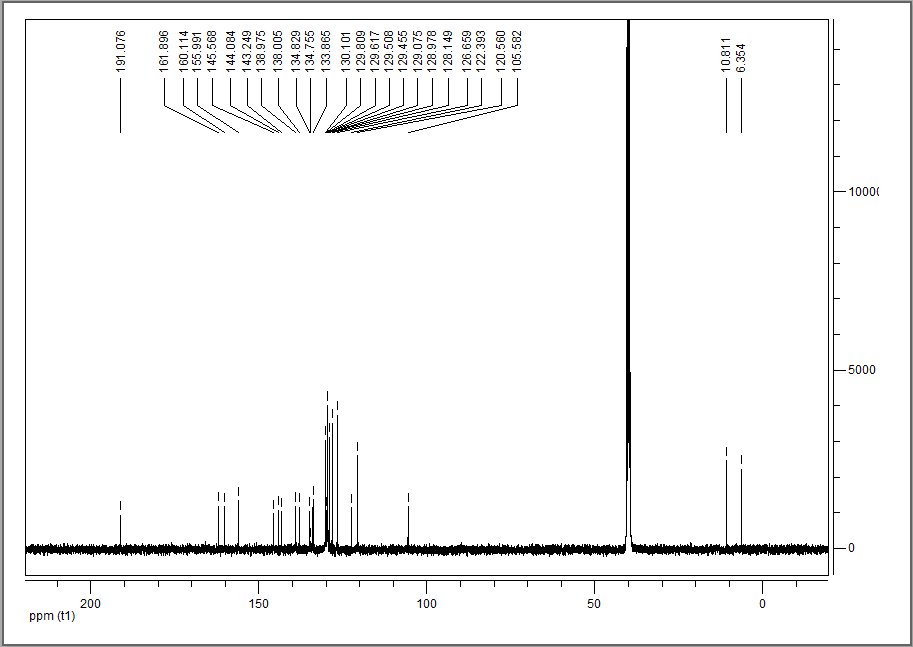


Figure S16: ^13^C NMR spectrum of compound ***6g***


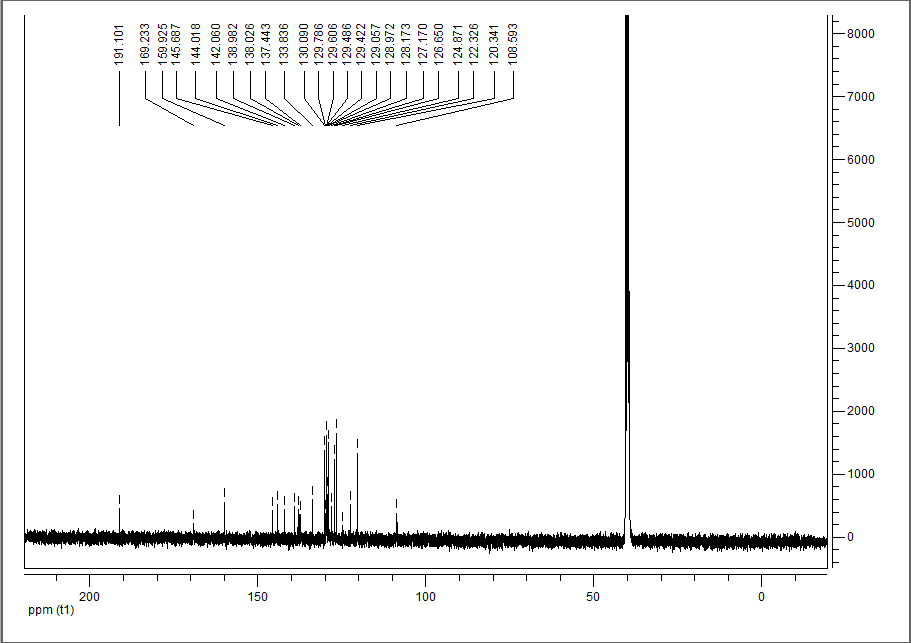


Figure S17: ^13^C NMR spectrum of compound ***6h***


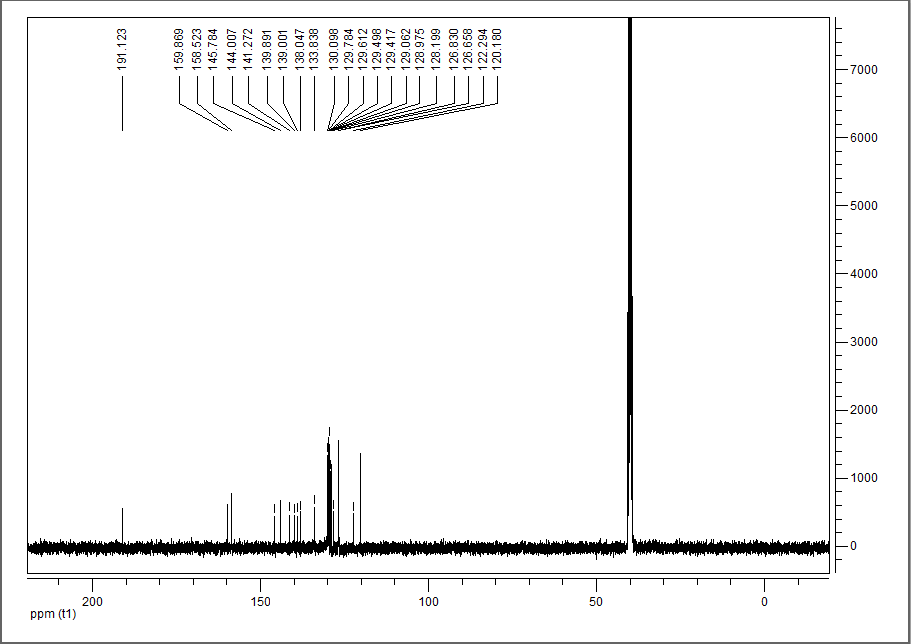


Figure S18: ^13^C NMR spectrum of compound ***6i***

**Mass Spectra of compounds *6a-i***


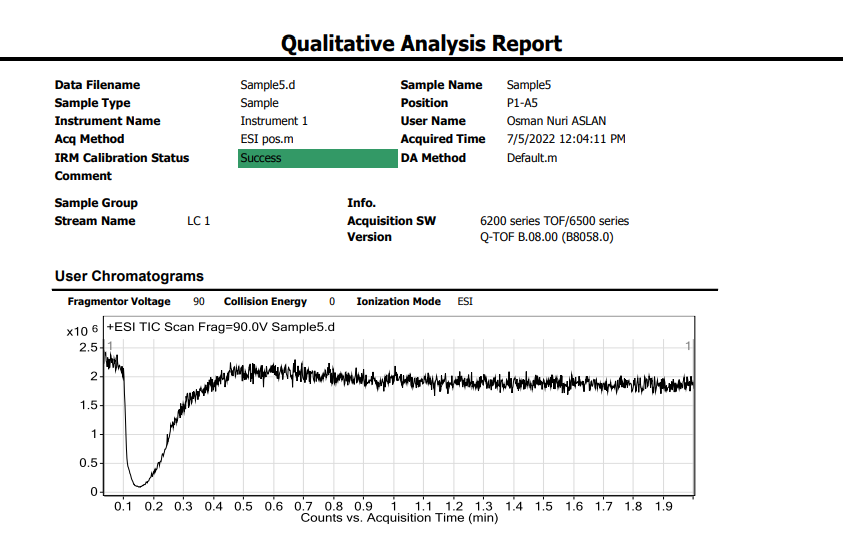


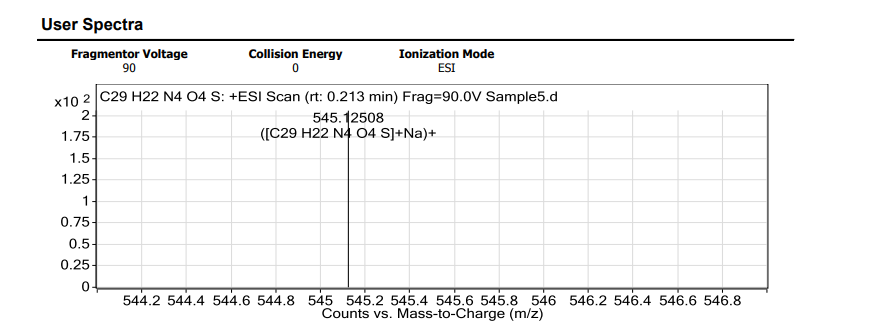


Figure S19: Mass spectra of compound ***6a***


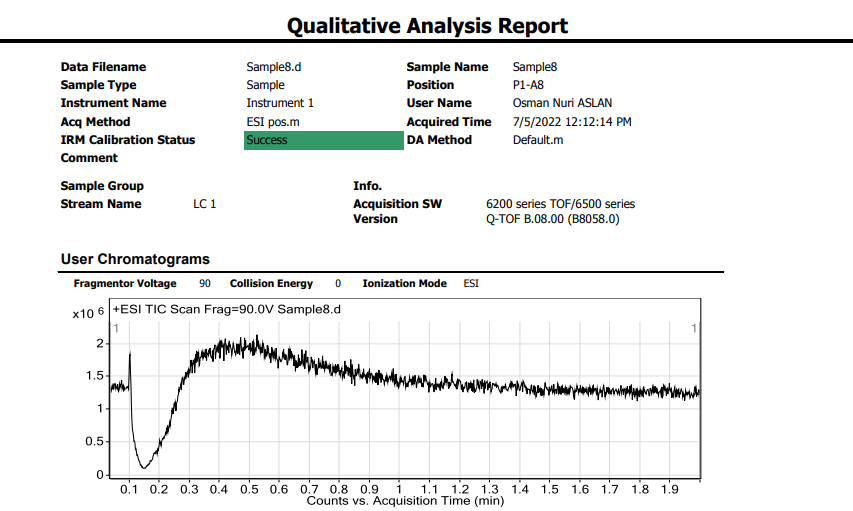


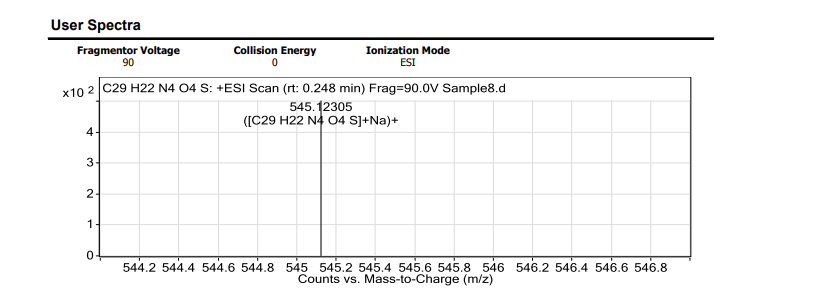


Figure S20: Mass spectra of compound ***6b***


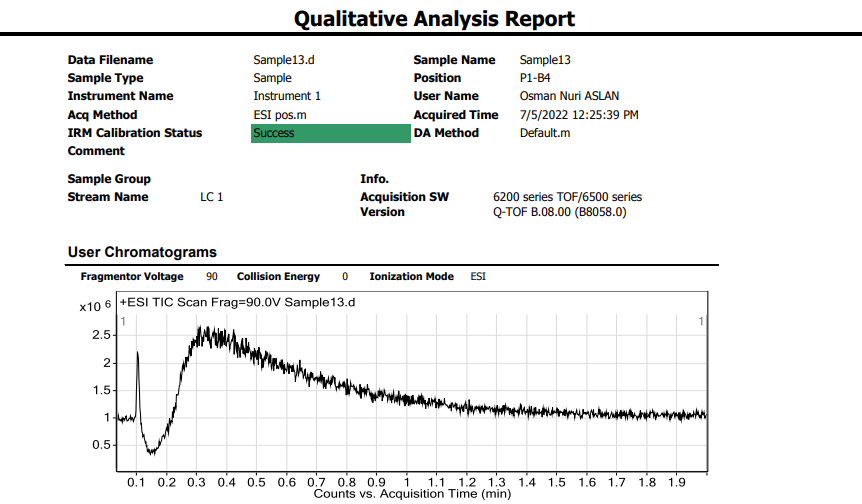


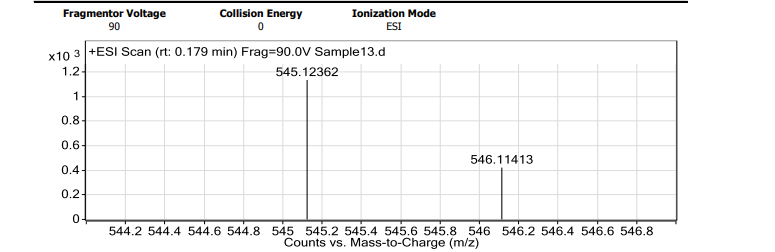


Figure S21: Mass spectra of compound ***6c***


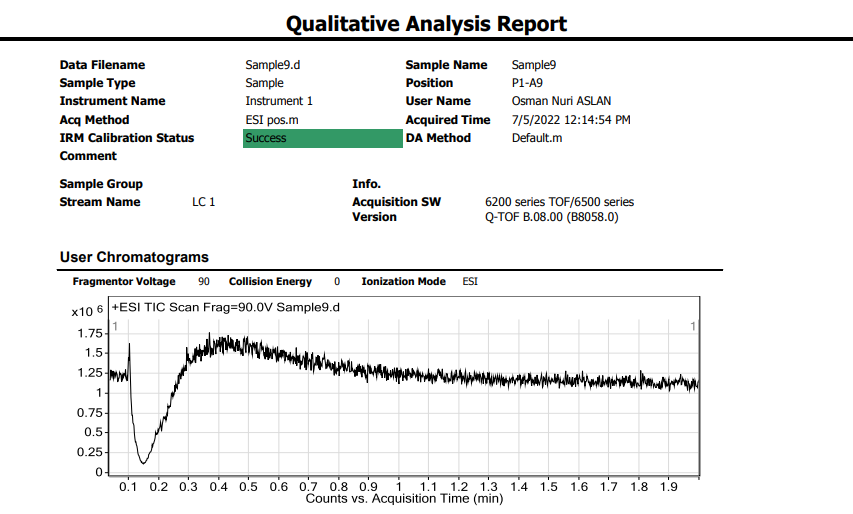


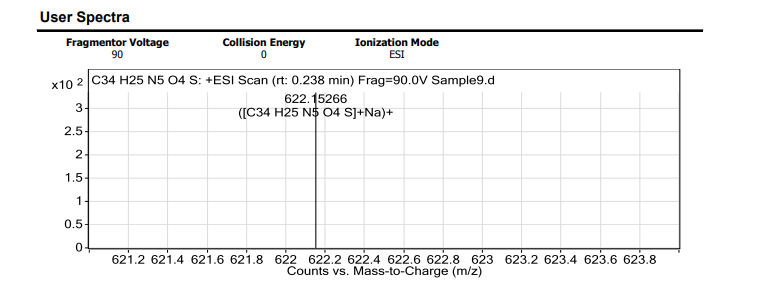


Figure S22: Mass spectra of compound ***6d***


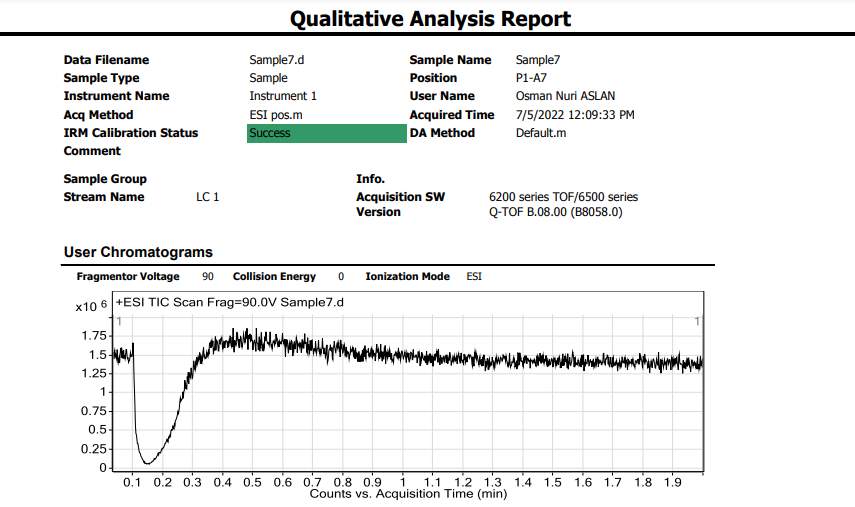


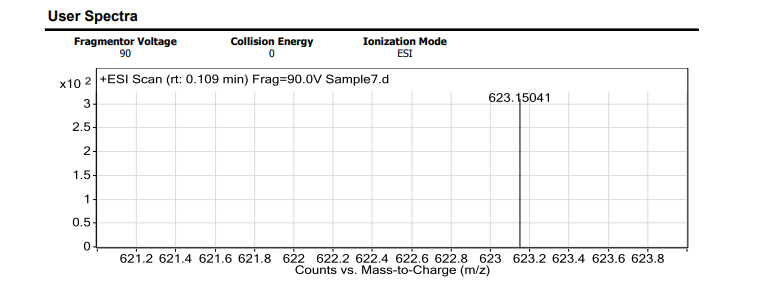


Figure S23: Mass spectra of compound ***6e***


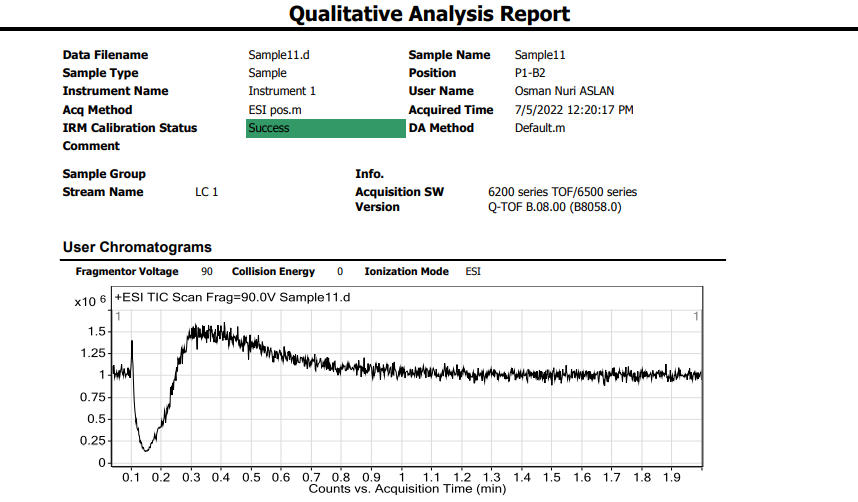


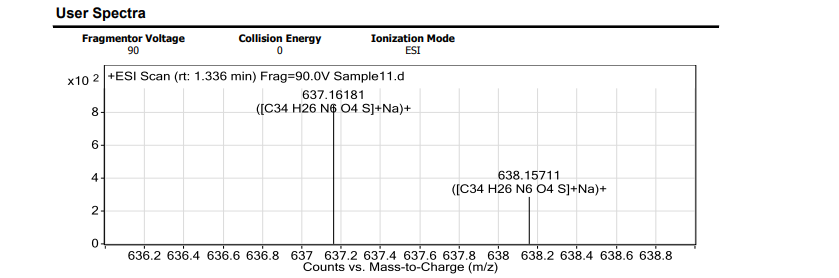


Figure S24: Mass spectra of compound ***6f***


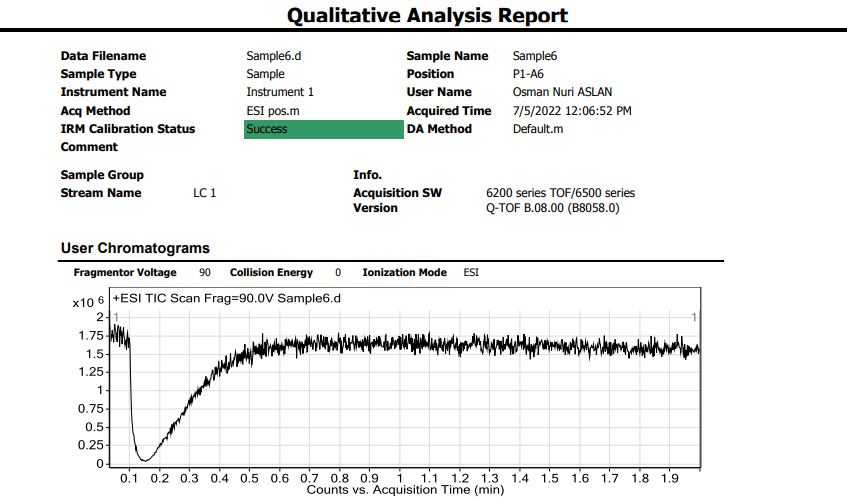


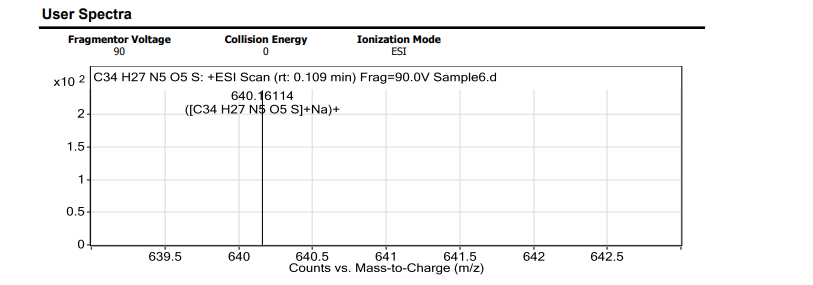


Figure S25: Mass spectra of compound ***6g***

,
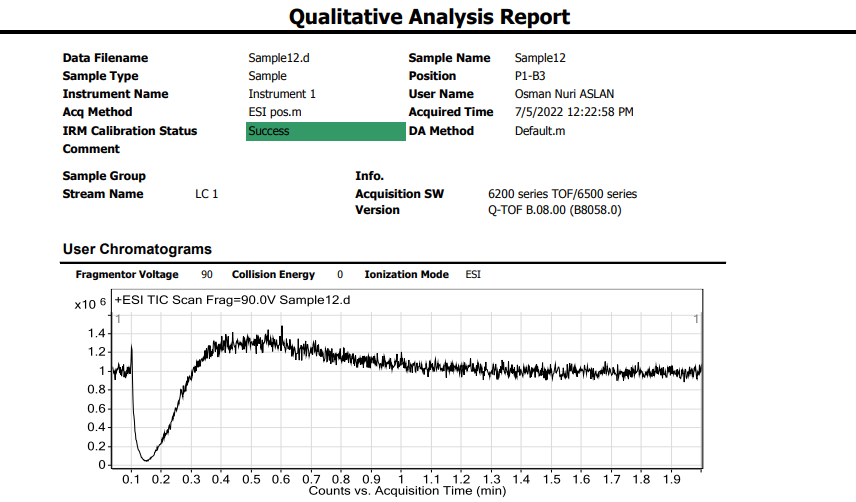


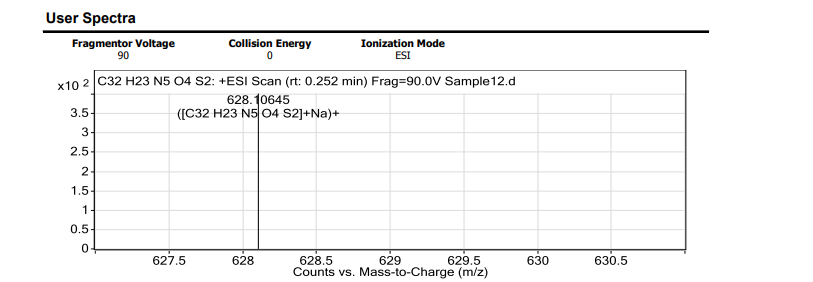


Figure S26: Mass spectra of compound ***6h***


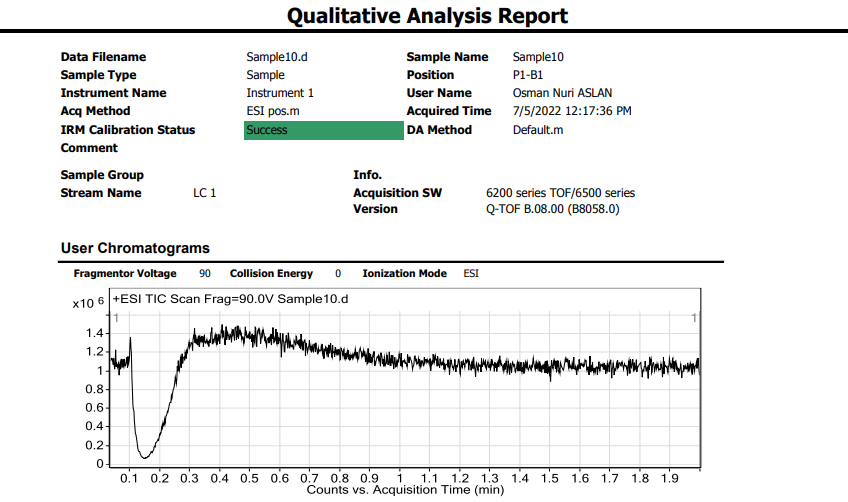


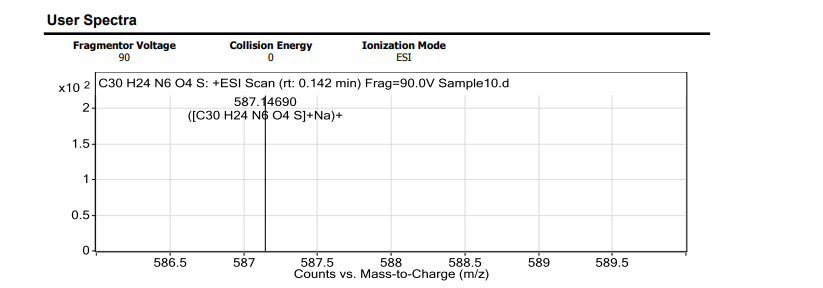


Figure S27: Mass spectra of compound ***6i***

**Purification of carbonic anhydrase I and II isoenzymes from human erythrocytes**

Erythrocytes were purified from human blood. The blood samples were centrifuged at 1500 rpm for 20 min and plasma was removed. Later, red cells were washed with isotonic solution (0.9% NaCl), and the erythrocytes were hemolyzed with 1.5 volumes of ice-cold water. Cell membranes were removed by centrifugation at 4 °C, 20000 rpm for 30 min. The pH of hemolysate was adjusted to 8.7 with solid TRIS (tris(hydroxymethyl)aminomethane). The hemolysate was applied to affinity column (Sepharose^®^4B-L-tyrosine-*p*-aminobenzene sulfonamide) pre-equilibrated with 25.0 mM TRIS-HCl/0.1 M Na_2_SO_4_ (pH 8.7). After extensive washing with a solution of 25.0 mM TRIS-HCl/22.0 mM Na_2_SO_4_ (pH 8.7), the hCA I and hCA II isoenzymes were eluted with the solution of 1.0 M NaCl/25.0 mM Na_2_HPO_4_ (pH 6.3) and 0.1 M NaCH_3_COO/0.5 M NaClO_4_ (pH 5.6), respectively. For quantitative protein determination, the Bradford method was used with bovine serum albumin as standard. Also, the purity control of the isoenzymes was performed with SDS-PAGE after the purification [46–52].

**Determination of hydratase and esterase activities of hCA I and hCA II**

The CO_2_ hydratase activity of the enzyme was determined at ~0 °C in a veronal buffer (pH 8.15) with the pH-stat method as the indicator and saturated carbon dioxide solution as the substrate in a final volume of 4.2 mL. The time (in seconds) taken for the solution to change from pH 8.15 to pH 6.50 was measured. The enzyme unit (EU) is the enzyme amount that reduces the non-enzymatic reaction time by 50%. The activity of an enzyme unit was calculated by using the equation ((*t_0_-t_c_*)*/t_c_*), where *t_0_* and *t_c_* are times for pH change of the non-enzymatic and enzymatic reactions, respectively [46–49].

Esterase activity was examined by following the change in the absorbance at 348 nm of 4-nitrophenylacetate to 4-nitrophenolate ion over a period of 3 min at 25 °C using a spectrophotometer (SHIMADZU UV 1700 PharmaSpec) according to the method described in the literature. The enzymatic reaction, in a total volume of 3.0 mL, contained 1.4 mL of 0.05 M TRIS–SO_4_ buffer (pH 7.4), 1.0 mL of 3.0 mM 4-nitrophenylacetate, 0.5 mL H_2_O and 0.1 mL enzyme solution. A reference measurement was obtained by preparing the same cuvette without enzyme solution [46–49, 53].

**Determination of IC_50_ and *K*_i_ values of the compounds**

To determine the IC_50_ values (the concentration of inhibitor producing a 50% inhibition of CA activity) of the compounds (***6a*–*i***), esterase activities of CA isoenzymes were examined in the presence of various inhibitor concentrations. Regression analysis graphs were drawn by plotting the percent enzyme activity versus inhibitor concentration and IC_50_ values were calculated [46–49].

To determine the *K*_i_ values as well as the inhibition type, three different inhibitor concentrations giving 30%, 50%, and 70% inhibition were selected. For each of these inhibitor concentrations, enzyme activity was measured in the presence of various substrate concentrations (0.3 mM, 0.4 mM, 0.5 mM, 0.6 mM and 0.7 mM) and the data were linearized with Lineweaver–Burk plot for *V*_max_ and the *K*_i_ determination. Enzyme activity was also measured in the presence of the same substrate concentrations but in the absence of any inhibitor to determine the *V*_max_ [46–49,53,54].

**Statistical analysis**

All the presented data were confirmed with three independent experiments and were expressed as the mean ± standard deviation (SD). Data were analyzed by using a one-way analysis of variance for multiple comparisons (SPSS 13.0, SPSS Inc., Chicago, IL). *p* < 0.0001 was considered to be statistically significant.
